# Deep history of an ancient subterranean crustacean: origins of the *Bathynellacea* (*Syncarida*)

**DOI:** 10.1098/rspb.2026.0943

**Published:** 2026-10-28

**Authors:** Shabuddin Shaik, Praveen Karanth

**Affiliations:** Centre for Ecological Sciences, Indian Institute of Science,Bangalore, Karnataka 560012, India

**Keywords:** bathynellaceans, biogeography, Gondwana, Mesozoic, molecular dating, subterranean crustaceans, vicariance

## Abstract

Plate-tectonic-mediated vicariance is often invoked to explain global disjunct distributions. However, since the advent of molecular tools, particularly molecular dating, it has become apparent that in many of the taxa, dispersal post-continental break-up better explains their current distribution. Nevertheless, these taxa belong to younger radiations and are less suitable for deep-time biogeographic studies. Here, we target an ancient lineage of subterranean freshwater crustacean of the order *Bathynellacea* with limited dispersal ability and global distributions to investigate the role of plate-tectonic-mediated vicariance. We investigated the evolutionary relationships among bathynellaceans using phylogenetic analyses of nuclear and mitochondrial markers. For the first time, four outgroup fossils were used to calibrate the time tree to date the origin and significant diversification events of bathynellaceans. Our results reveal deep phylogenetic divergences among Bathynellacean families, suggesting an ancient origin dating back to the Mesozoic or possibly even the Late Palaeozoic. The early branching lineages are geographically distributed across former Gondwanan landmasses and Eurasia, supporting a dual origin scenario and challenging previous views of a single Laurasian ancestry. The taxonomic implications of our phylogeny are also discussed. Overall, this study demonstrates *Bathynellacea* as a valuable model for understanding deep-time evolutionary processes.

## 1. Introduction

The role of plate tectonics in shaping the geographic distribution of organisms has long been a central theme in historical biogeography. Following the widespread acceptance of Wegener's theory of continental drift in the 1960s, plate-tectonic-mediated vicariance became a widely invoked mechanism to explain global disjunct distributions in diverse groups [1–5]. However, evolutionary relationships among taxa in these studies were largely inferred from traditional morphology-based taxonomy. With the advent of molecular phylogenetic tools, particularly molecular dating, there has been increasing evidence supporting post-break-up long-distance dispersal as a major contributor to present-day distributions in many plant and animal groups [6–13]. According to the vicariance scenario, taxa originally distributed across the supercontinent Pangaea were separated onto different continents following tectonic fragmentation, leading to widespread disjunctions. In this regard, the Indian subcontinent is an interesting entity as it was part of Gondwana around 200 mya and post break-up of Gondwana, it merged with Eurasia around 35 mya [14,15]. This tectonic journey has led to the hypothesis that India acted as a vehicle for transporting ‘Gondwanan’ lineages into Asia [16,17].

In the Indian context, a recent review by Karanth [18] concluded that India's current tetrapod diversity was largely assembled through dispersal post break-up. Additionally, while the Late Cretaceous tetrapod fauna of India shows Gondwanan affiliation, much of this hypogeal fauna was obliterated owing to Cretaceous–Paleogene mass extinction exacerbated by climatic stress and volcanic activity. The review recommends targeting fossorial soil invertebrates to study plate-tectonic-mediated vicariance as they are shielded from extreme conditions on the surface and less likely to have gone extinct. In this regard, the subterranean fauna might also be an appropriate group as they are tied down to the landmass, are largely unaffected by above-ground changes and have long been recognized as suitable candidates for understanding the predominant historical biogeography process [19]. As such, they offer a valuable window into deep-time biogeographic processes that may no longer be detectable in surface-dwelling groups.

The subsurface aquatic animals, generally referred to as ‘subterranean fauna/stygofauna’ (also termed stygobionts) [20], are found in typical karst systems, alluvial deposits and fractured rock aquifers. Stygofauna comprise groups ranging from protozoans to fishes, with crustaceans accounting for 43% of their known diversity [21]. Bathynellaceans, together with Parastenocaridid copepods, are characteristic elements amongst crustacean stygobionts that are highly adapted to life in continental groundwater systems and are almost exclusively confined to such habitats [22]. Despite their narrow ecological niche, lack of active dispersal mechanisms [23,24] and the absence of resting stages for passive dispersal, these groups exhibit broad global distribution occurring on all continents except Antarctica [25,26]. This has led to considerable speculation regarding their biogeographic history. A prevailing hypothesis, supported by multiple studies, posits that their widespread distribution was established prior to the break-up of Pangaea [27–29]. While both groups are characteristic components of freshwater interstitial fauna [30], bathynellaceans, in particular, are considered one of the most ancient lineages of freshwater crustaceans whose ancestors inhabited the seas in the Carboniferous or even earlier [27]. Notably, they are now absent from the epigean realm. Their widespread geographic distribution is more convincingly explained by a vicariance model associated with the break-up of Pangaea [24,27,31], rather than classical long-distance dispersal. This makes them an ideal group for testing vicariance-based biogeographic hypotheses. However, to date, no comprehensive biogeographic studies have been undertaken on this group, mainly owing to a lack of molecular studies from certain continental plates such as India, although there is a pioneering work where two species from India are included with molecular data [32].

The present study attempts to address this gap by combining newly generated molecular data from India with available global data and conducting detailed phylogenetic and divergence analyses using fossils, to refine our understanding of the evolutionary history and biogeography of the *Bathynellacea* group. Though the phylogenetic framework is based on only two loci, the *18S* rRNA gene is a slowly evolving nuclear marker widely used in broad arthropod and crustacean phylogenies, and it tends to resolve very deep splits at family-, order- or even class-level [33,34].

Bathynellaceans are minute, vermiform, primitive eumalacostracan crustaceans, which are a typical component of the Indian subterranean habitats. The order *Bathynellacea* comprises three families: *Bathynellidae Grobben*, 1905, *Parabathynellidae* [35] and a third family, *Leptobathynellidae* [35], was recently reaffirmed its taxonomic validity through molecular analyses [32]. Globally, the family *Bathynellidae* currently includes 114 species across 37 genera [36,37], *Parabathynellidae* comprises approximately 230 species in 45 genera [37,38] and *Leptobathynellidae* is represented by 18 species across seven genera.

In India, the order *Bathynellacea Chappuis*, 1915, is represented by all three recognized families. The family *Parabathynellidae* comprises 23 species across three genera: *Habrobathynella* [39] (17 spp.), *Chilibathynella* Noodt, 1963 (1 sp.) and *Atopobathynella* [39] (5 spp.) [40]. The family *Leptobathynellidae* is represented by a single genus, *Parvulobathynella* [39] with three known species. It is to be noted that all the members of *Parabathynellidae* and *Leptobathynellidae* so far reported from India are from the peninsular region, which, according to Mani [15, p. 700], constitutes ‘biogeographically India vera’, the largest and the oldest region of differentiation of the original floras and faunas of India. These two families therefore might reflect a typical Gondwanan heritage. By contrast, the family *Bathynellidae* shows comparatively lower diversity in India, with only five species reported in the following three endemic genera: *Camachobathynella* Ranga Reddy, Shabuddin and Totakura, 2015 (1sp.)*, Indobathynella* Ranga Reddy and Totakura, 2012 (2 spp.) [41], *Serbanibathynella* Ranga Reddy and Schminke, 2005 (2 spp.) [42]. These genera are not known to occur outside India and exhibit distinct biogeographic patterns.

The *Bathynellacea* group has no known fossil record and extant representatives exhibit global distribution excluding Antarctica. The ancient origins of *Bathynellacea* prompted several workers to hypothesize that this group must date to at least of Pangaean times [27]. Schminke [27] initially proposed that certain groups of parabathynellid genera (*Cteniobathynella* and *Chilibathynella*) followed distinct Eurasian and Gondwanan tracks, suggesting a potential dispersal from an East Asian as centre of origin. However, Schminke [30] revised his stance with Schram [31], and agreed that bathynellaceans probably originated from a Paleozoic, tropical Laurentian ‘minicarid’ stock, with subsequent outward dispersal slowly to northern and southern landmasses following the formation of Pangaea in the Permian before it broke apart (figure 1). The presence of closely related but geographically isolated taxa in Europe, Asia and North America substantiates this hypothesis. Until now, no attempts have been made to test this hypothesis owing to the lack of a direct fossil record. In this study, we aim to evaluate the evolutionary origin of *Bathynellacea* by including samples from India and incorporating fossil calibrations from closely related taxa (see supplementary material on the systematic controversy of *Bathynellacea*). Specifically, we investigate whether the estimated dates of the basal divergences within the groups overlap with the time period of the break-up of Pangaea.

**Figure 1.**
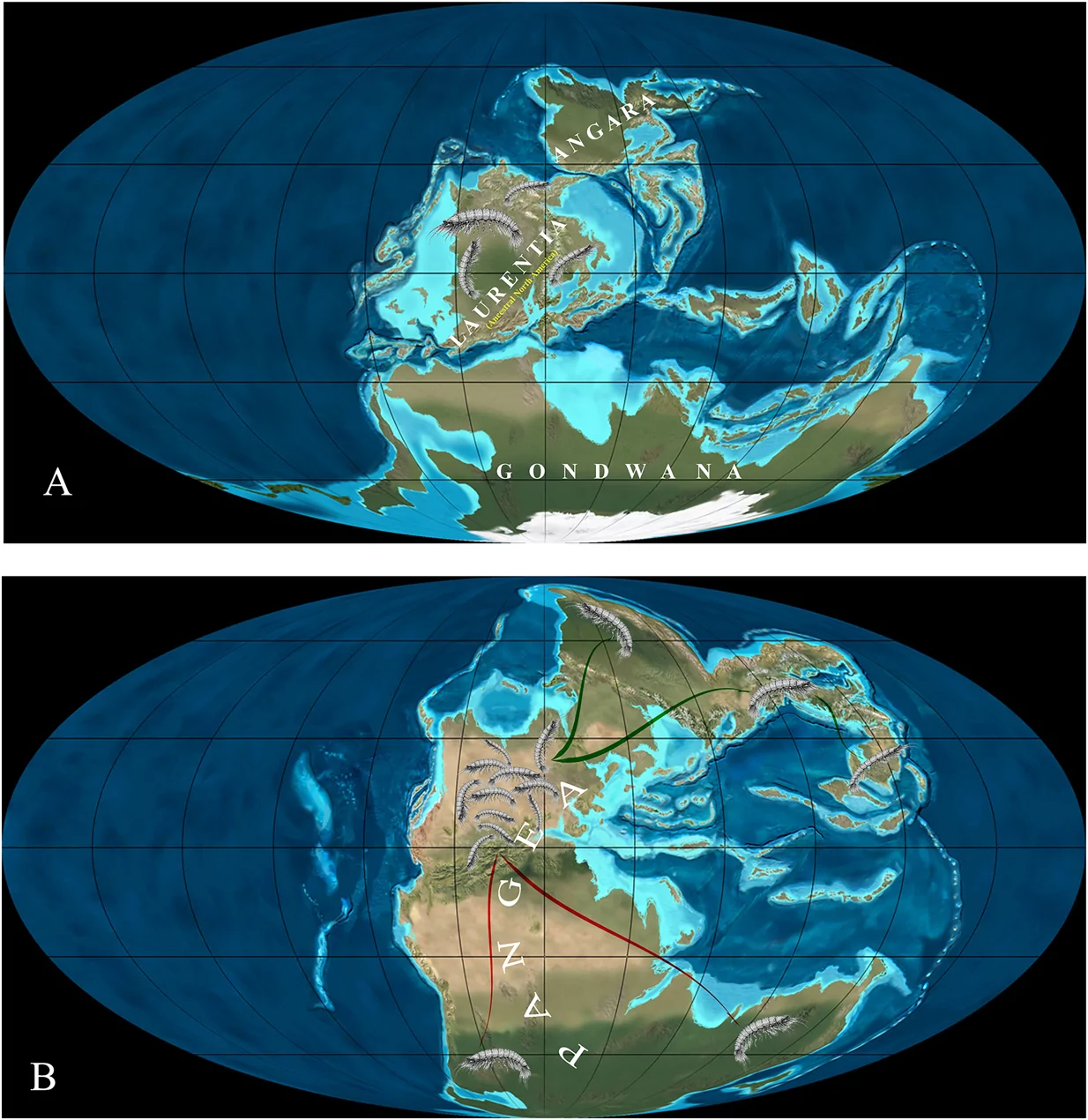
Hypothesized biogeographic scenario [31], illustrating the dispersal of the ancestral lineage prior to the break-up of Pangaea and subsequent divergence to Laurasia (green) and Gondwana (red). Arrows indicate inferred migration routes of ancestral populations: (A) plate tectonic map of Early Carboniferous (Mississippian; 340 Ma); (B) plate tectonic map of Middle Triassic (Ladinian; 240 Ma).

## 2. Material and methods

Approximately 300 samples (2022–2024) were collected from the Krishna–Godavari Basin (figure 2), yielding 117 specimens for morphological and molecular analyses. Partial *18S* and *COI* genes were sequenced, validated and combined with published data for Maximum Likelihood (ML) and Bayesian methods, showing no mito-nuclear discordance. Divergence times were estimated in BEAST [43] using four fossil calibrations. Prior–posterior comparisons and Kullback–Leibler (KL) divergence were used to assess calibration influence and molecular signal contribution (for more details: see supplementary material available at (doi:10.6084/m9.figshare.c.8680232).

**Figure 2.**
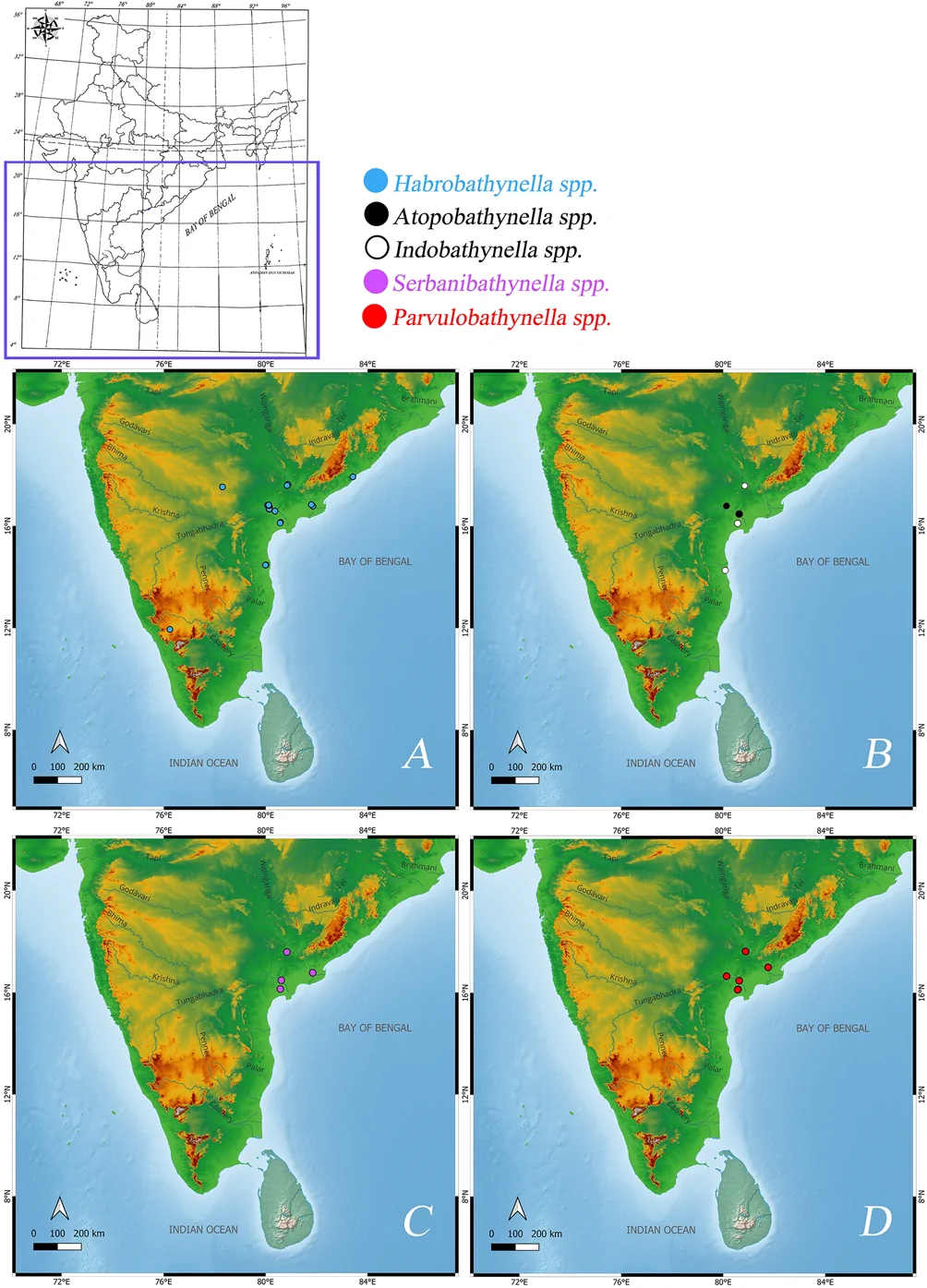
Map showing the sampling localities of Indian Bathynellacean species included in this study: (*A*) *Habrobathynella* spp. (sky blue); (*B*) *Atopobathynella* spp. (black) and *Indobathynella* spp. (white); (*C*) *Serbanibathynella* spp. (purple) and (*D*) *Parvulobathynella* spp. (red).

## 3. Results

### (a) Phylogenetic analyses

A total of 83 new sequences were generated in this study, including 45 sequences for the *18S* rRNA gene (PX832242–PX832286) and 38 sequences for the *COI* gene (PX831691–PX831728). The final concatenated dataset consisted of 3417 base pairs with gaps from 171 taxa, i.e. tips in the phylogeny (124 downloaded from GenBank and 47 generated in this study), representing all biogeographic realms except the Neotropical realm (figure 3). This study significantly contributes to the global phylogeny of *Bathynellacea*, particularly for the two genera of the *Bathynellidae* family from India, namely *Serbanibathynella* and *Indobathynella*, which were not previously represented in phylogenetic analyses. All available sequences from GenBank were included, along with newly generated sequences from India. The phylogenetic analyses conducted using ML and Bayesian inference (BEAST [43]) yielded largely congruent topologies (figures 4 and 5). In both the ML and Bayesian inference (BI) trees, the families *Parabathynellidae* and *Bathynellidae* were not monophyletic. Across all three families, eight major clades (C1–C8) were consistently recovered with strong Bayesian support (posterior probability (*PP*) = 1.0). Clades C1–C4 clustered together and consisted of most of the genera in the family *Parabathynellidae*.

**Figure 3.**
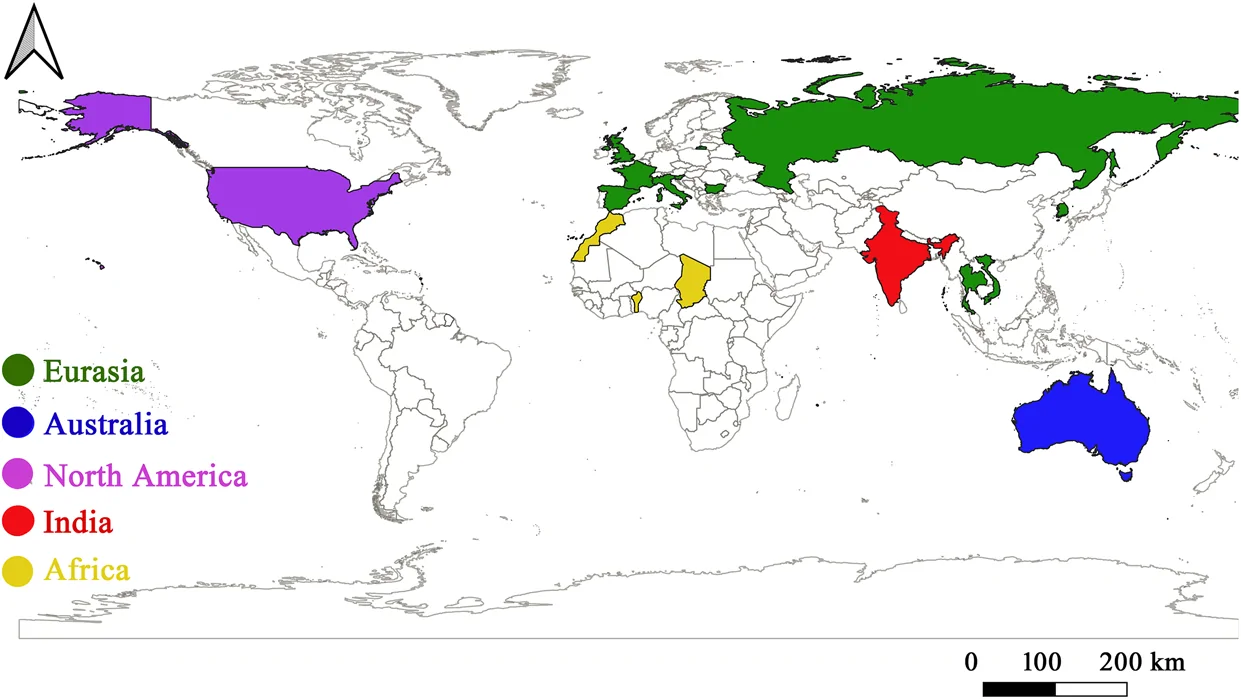
Global map showing the geographic distribution of the countries from which the *Bathynellacea* taxa were sampled (including published data from [32]). Colours indicate the continental categories used throughout the analyses.

**Figure 4.**
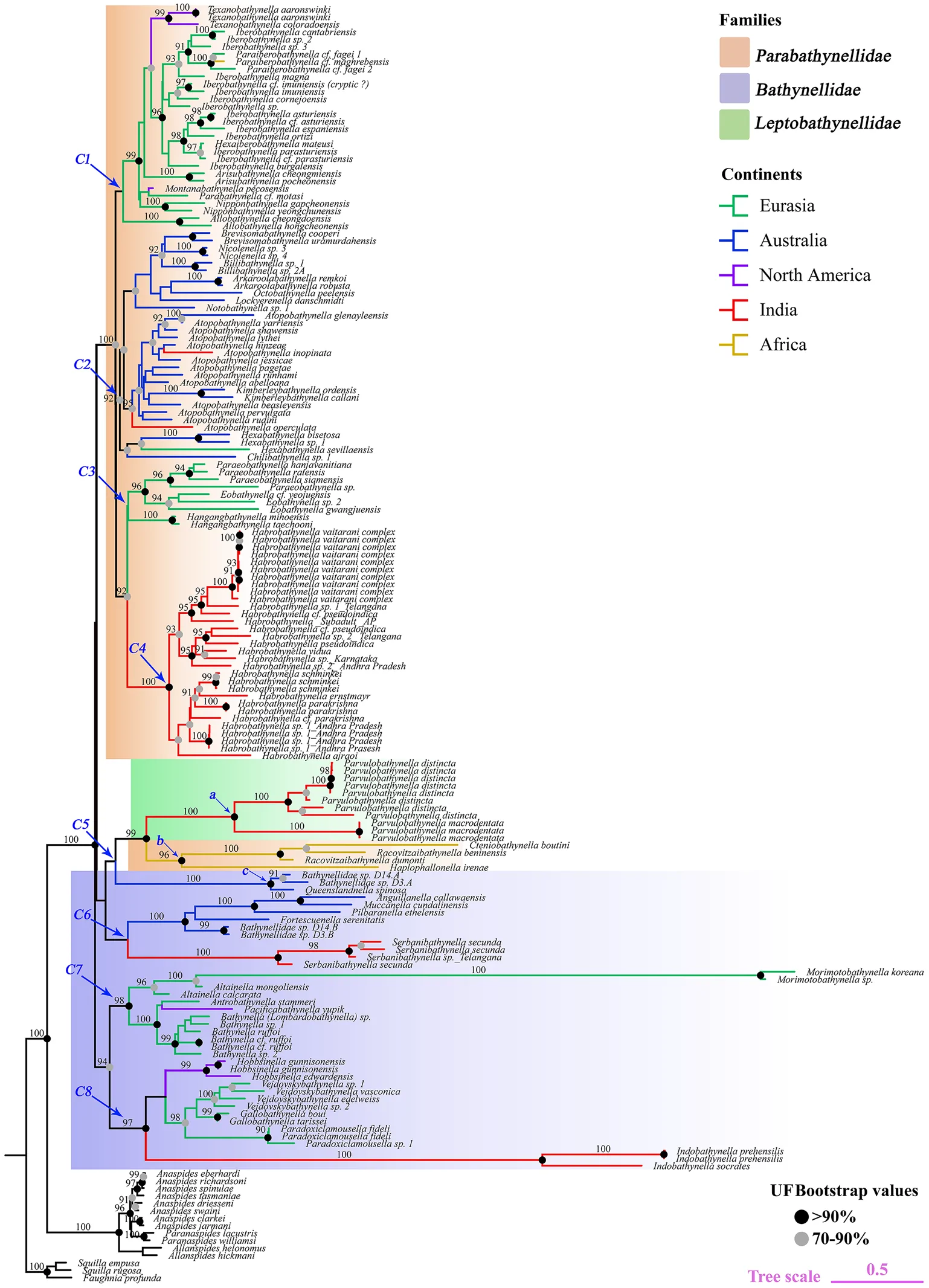
Maximum likelihood (ML) tree constructed using *COI* and *18S* markers implemented in IQ-TREE [44]. Circles represent ultrafast bootstrap and numerical values represent *SH-aLRT* support. UF Bootstrap and *SH-aLRT* values below 70 and 90, respectively, are not shown here. C1, C2, etc., represent clades and a, b, c represent sub-clades of C5.

**Figure 5.**
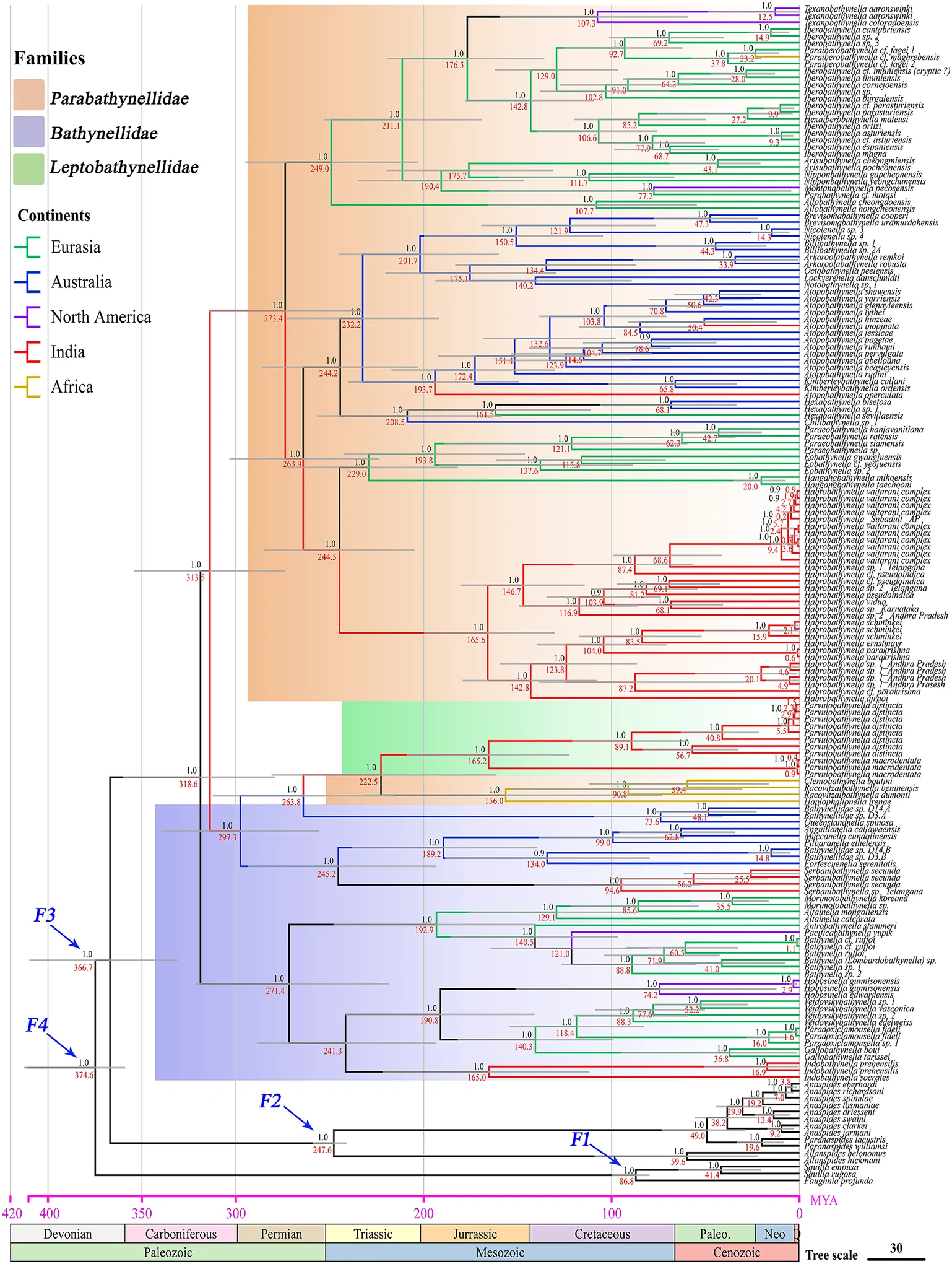
Divergence times inferred using BEAST. Arrows indicate the positions of the four fossil calibrations employed in this study (see supplementary material, table S1). Node ages are shown in red font and black values denote posterior probabilities (>0.9). The 95% highest posterior density (HPD) intervals are depicted as horizontal bars at each node, indicating the range of credible divergence times.

The first clade (C1) (*PP* = 1.0, *UFBoot* = 54, *SH-aLRT* = 65) included predominantly Laurasian genera, *Texanobathynella*, *Iberobathynella*, *Paraiberobathynella*, *Hexaiberobathynella*, *Arisubathynella*, *Montanabathynella*, *Parabathynella*, *Nipponbathynella*, *Allobathynella* and one North African taxon, *Paraiberobathynella* cf. *maghrebensis*. The topology of this clade is consistent with previous molecular phylogenetic studies [32,45]. In both trees, the South Korean genus *Allobathynella* was recovered as early diverging lineage within this clade. The second clade (C2) (*PP* = 1.0, *UFBoot* = 92, *SH-aLRT* = 75) included the cosmopolitan genus *Hexabathynella*, along with Australian and Indian genera, namely *Brevisomabathynella*, *Nicolenella*, *Billibathynella*, *Arkaroolabathynella*, *Octobathynella*, *Lockyerenella*, *Notobathynella*, *Atopobathynella*, *Kimberleybathynella* and *Chilibathynella*. Clade C3 is strongly supported by Bayesian analysis (*PP* = 1) but weakly in the ML tree (*UFBoot* = 55, *SH-aLRT* = 69), and included two Korean genera (*Eobathynella* and *Hangangbathynella*) and one Southeast Asian genus (*Paraeobathynella*). The last well-supported clade (C4) (*PP* = 1.0, *UFBoot* = 100, *SH-aLRT* = 100) composed of Indian species of *Habrobathynella*, which was sister to clade C3.

The family *Leptobathynellidae* (Clade C5a) was monophyletic, and comprised exclusively of Indian taxa (*Parvulobathynella* spp.). This clade was sister to a clade (C5b) of African genera (*Cteniobathynella*, *Racovitzaibathynella* and *Haplophallonella*), which are currently assigned to the *Parabathynellidae* family (see taxonomic implications). This Indian–African clade is strongly supported (*PP* = 1.0; *UFBoot* = 95; *SH-aLRT* = 99) and, in turn, was sister to the subfamily Queenslandbathynellinae (*Bathynellidae* (C5c)), with strong Bayesian support (*PP* = 1.0) but weaker ML support (*UFBoot* = 56; *SH-aLRT* = 76).

Within the remaining *Bathynellidae*, the clade C6 is represented by four described Australian genera (*Anguillanella, Muccanella*, *Pilbaranella* and *Fortescuenella*), together with two unidentified taxa (*Bathynellidae* sp. D3.B and D14.B). As noted by Perina *et al.* [46], this group may represent a provisional subfamily, ‘Austrobathynellinae?’, though confirmation of its monophyly will require molecular data from its type species (*Austrobathynella patagonica* Delamare Deboutteville, 1960). This Australian clade was recovered as the sister to the Indian genus *Serbanibathynella*, with strong Bayesian support (*PP* = 1.0) but weak ML support (*UFBoot* = 63, *SH-aLRT* = 85).

The clade C7 corresponded to the subfamily Bathynellinae (*PP* = 1.0, *UFBoot* = 96, *SH-aLRT* = 98), and included predominantly Laurasian genera such as *Morimotobathynella*, *Altainella*, *Antrobathynella*, *Pacificabathynella* and several species of *Bathynella* (*PP* = 0.9, *UFBoot* = 91, *SH-aLRT* = 98) from Europe (Bulgaria, Slovenia and Italy). The clade C8 (*PP* = 0.9, *UFBoo*t = 98, *SH-aLRT* = 97) included representatives of the subfamily Gallobathynellinae and *Indobathynella* taxon from India. The Gallobathynellinae (excluding *Indobathynella*) composed of the North American genus *Hobbsinella* and the Eurasian genera, including *Vejdovskybathynella*, *Gallobathynella* and *Paradoxicamousella*.

### (b) Divergence time estimation

The time-calibrated phylogeny suggests that the basal divergence among the three Bathynellacean families: *Parabathynellidae*, *Bathynellidae* and *Leptobathynellidae* occurred during the Carboniferous period (approx. 318 mya; 95% HPD: 279–360 mya), which is substantially older than previous estimates, placing these divergences in the Triassic [32], but still congruent with the broader range (Pre-Pangaea), as mentioned by Camacho [47] and [48]. The *Leptobathynellidae*, which is nested within clade C5 together with the Queenslandbathynellinae, diverged from clade C6 (Austrobathynellinae + *Serbanibathynella*), during the Late Permian at approx. 297 mya (255–339 mya). The subfamilies Bathynellinae (C7) and Gallobathynellinae (C8), along with the Indian taxon *Indobathynella*, diverged later in the Middle Permian (approx. 271 mya; 218–321 mya). The final divergence of *Indobathynella* from its Laurasian relatives occurred approximately 241 mya (193–288 mya), corresponding to the Middle Triassic.

The most recent common ancestor for *Parabathynellidae* was estimated to the Permian (approx. 273 mya; 233–314 mya). The South Korean genus *Allobathynella* (clade C1) diverged early from the rest of the taxa within *Parabathynellidae* (approx. 249 mya; 203–295 mya), followed by *Habrobathynella* genera during the Late Triassic (approx. 244 mya; 205–285 mya), suggesting an early divergence within the family. The major diversification of *Habrobathynella*, *Parvulobathynella* and *Indobathynella* occurred in a similar time period, during the Middle Jurassic (Callovian stage, approx. 165 mya), while *Serbanibathynella* represents a comparatively recent lineage, dating to the upper Cretaceous (Cenomanian stage; approx. 94 mya)

### (c) Comparing Prior and Posterior Distributions

Comparison of the prior and posterior marginal age distributions (figure 6) revealed node-specific differences in distributional shape, indicating a variable influence of molecular data across the four calibrated nodes examined in this study. For Syncarida_F1, prior and posterior distributions differ in shape, with the posterior exhibiting greater density toward older ages and an overall broader distribution. This pattern indicates that molecular data modify the age distribution for this node without entirely overriding fossil-based expectations. For Anaspides_F2, the posterior distribution exhibits an increased density peak relative to the prior, with probability mass concentrated toward the younger end of the calibrated range. Although the posterior remains broadly overlapping with the prior, the altered distributional shape suggests an influence of molecular data on divergence-time estimation at this node, resulting in a more sharply defined age distribution.

**Figure 6.**
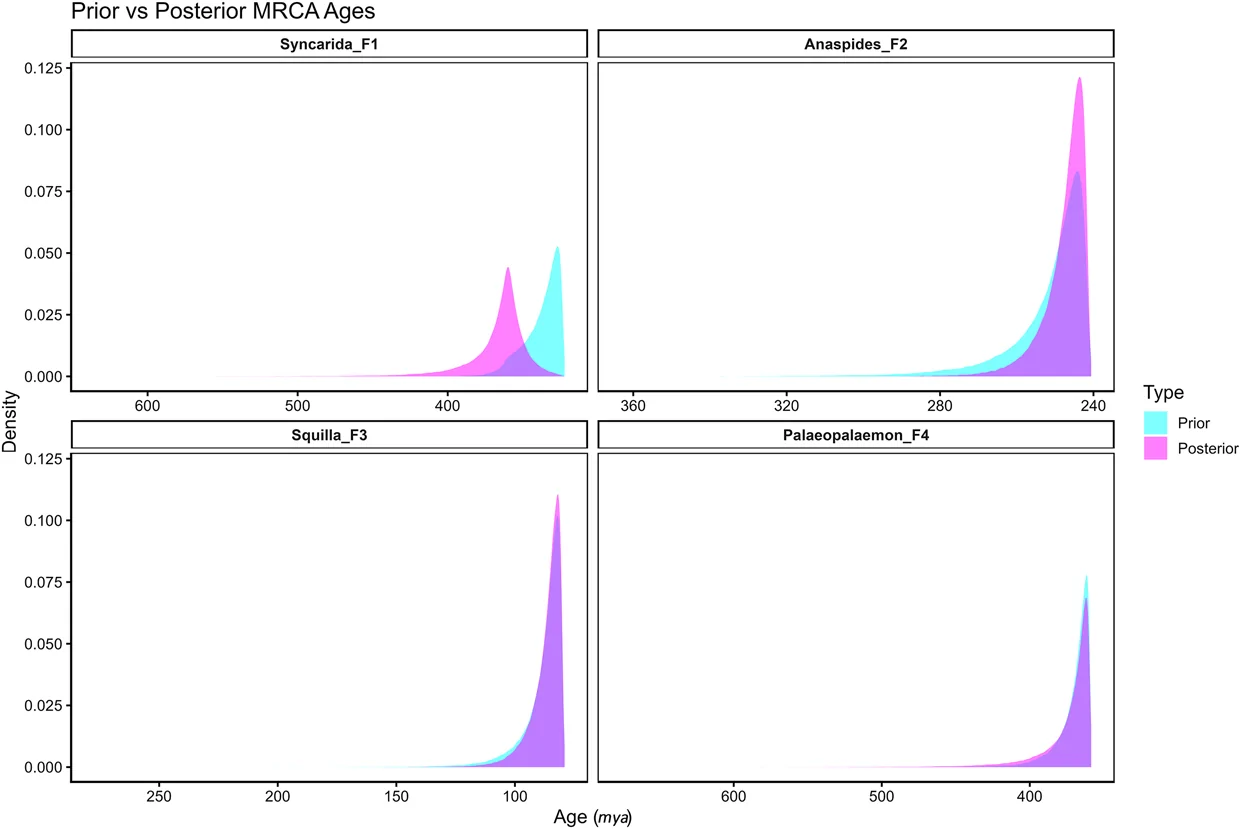
Comparison of marginal prior (cyan) and posterior (pink) age distributions for the four fossil-calibrated nodes, illustrating the influence of prior assumptions and molecular data.

By contrast, Squilla_F3 shows extensive overlap between prior and posterior distributions, with only minor changes in peak height and overall shape. This congruence suggests that divergence-time estimates for this node are largely constrained by the fossil calibration, with limited additional temporal signal contributed by molecular data. Similarly, Palaeopalaemon_F4 exhibits strong concordance between prior and posterior distributions, with nearly identical shapes and peak positions. This pattern suggests that fossil calibration has a dominant influence on the estimated age of this node, with minimal modification by molecular evidence. Consistent with these patterns, KL divergence analyses indicated a moderate departure of posterior distributions from their corresponding priors (KL = 0.336), reflecting detectable information gain from molecular data.

## 4. Discussion

Stygofauna serve as an excellent model system for testing plate-tectonic-mediated vicariance owing to their subterranean lifestyle, which limits their dispersal and effectively ties them to specific landmasses. Their isolation from surface-level environmental changes also makes them more probably to have survived past mass extinction events. One such group is the *Bathynellacea*, a lineage of subterranean crustaceans found on all continents except Antarctica. In this study, we reconstructed the global phylogeny and time tree of bathynellaceans for the first time based on outgroup fossils by addressing a major sampling gap and generating new genetic data from the Indian plate, one of the most tectonically dynamic fragments of Gondwana. The biogeographic and taxonomic implications of this study are discussed below.

Among the three families of Bathynellaceans Grobben, 1905, *Leptobathynellidae* was recovered as monophyletic, whereas *Bathynellidae* was not monophyletic, as reported by Camacho *et al.* [32]. In addition, our analyses recovered *Parabathynellidae*, as non-monophyletic. The family *Parabathynellidae* consisted of multiple clades largely confined to specific geographical areas. The first clade (C1), constituted taxa that have a predominantly Laurasian distribution and included *Texanobathynella*, *Hexaiberobathynella*, *Iberobathynella*, *Paraiberobathynella*, *Arisubathynella*, *Montanabathynella*, *Parabathynella* and *Nipponbathynella*. The inclusion of North African representatives (*Paraiberobathynella* cf*. maghrebensis*) in the Laurasian clade may reflect a Late Eocene to Miocene dispersal via the Rif–Betic land connections between Iberian Peninsula (part of Europe) and Morocco (part of Africa) during tectonic convergence. The date of these events coincides with diversification between African and Eurasian *Paraiberobathynella* (23 mya; 11–38 mya). Further sampling across North Africa and Southern Europe, combined with additional genetic markers, would help clarify whether this pattern reflects true historical connectivity or a case of taxonomic misidentification (see §5) or cryptic species.

The second clade (C2), composed of the cosmopolitan genus *Hexabathynella* along with several Australian and Indian genera, including *Brevisomabathynella*, *Nicolenella*, *Billibathynella*, *Arkaroolabathynella*, *Octobathynella*, *Lockyerenella*, *Notobathynella*, *Atopobathynella*, *Kimberleybathynella* and *Chilibathynella*, suggests a historically widespread lineage with deep evolutionary roots. The high support values obtained from ML and BEAST analyses, combined with the current distribution of the taxa, suggest a possible Gondwanan origin of this clade. It is noteworthy to mention here that the genus *Hexabathynella* is the only cosmopolitan taxon among all known genera of *Bathynellacea*, with 28 described species [38]. Camacho [49] found that the occurrence of the species with the fewest apomorphic traits (*Hexabathynella paranaensis*) in brackish water supports the hypothesis of a marine ancestry for the genus. Schminke [27] invoked euryhalinity, a key biological trait that might have facilitated its broad distribution. This euryhaline capacity is rare among bathynellaceans; most of them are obligate freshwater stygobionts with limited dispersal capability. This physiological flexibility probably enabled *Hexabathynella* to exploit coastal, estuarine or even marine interstitial habitats, thereby opening new dispersal routes that were not utilizable by the rest of the genera. During periods of lowered sea level, such dispersal could have occurred through coastal corridors across continents and thus may suggest that oceanic dispersal played a role alongside traditional vicariant models. It is also plausible that some euryhaline species remained in their original habitat and marine transgressions/regressions were responsible for their subsequent dispersal, and local vicariant phenomena [49] led to the current distributions and short-range endemism.

It is hypothesized that ancestral *Hexabathynella* populations occupied Pangaean coastal regions during the Permian (250–290 mya), subsequently expanding into inland freshwater habitats prior to the continental break-up in the Mesozoic. Interestingly, despite being cosmopolitan, no species of *Hexabathynella* has been reported from India so far, reflecting gaps in sampling or probably true absence. Five new species have been described from Australia in recent years, confirming the monophyly of the genus using a three-gene tree, but not by a two-gene tree [38,50,51].

The third and fourth clades (C3 and C4) were recovered as sister lineages. These clades comprise Asian taxa (*Paraeobathynella*, *Eobathynella* and *Hangangbathynella*) and Indian taxa (*Habrobathynella*). Overall, the four clades of family *Parabathynellidae* are largely confined to various fragments of Gondwana and Laurasia. Clades C1 and C2 are distributed primarily in Laurasia (Eurasia + North America) and Gondwana (Australia + India), respectively. Similarly, clades C3 and C4 are distributed in Laurasia (Eurasia) and Gondwana (India), respectively. The divergence among these clades spans a time period from 244 to 273 mya, coinciding with early tectonic differentiation of the Pangaean supercontinent. Thus, it appears that regional tectonic structuring (regional tectonism) within Pangaea, rather than continental break-up per se, might have shaped the present-day distributions of family *Parabathynellidae*. Therefore, ancestral range structuring predated continental fragmentation and was subsequently reinforced by Late Mesozoic continental break-up.

The family *Bathynellidae* is not monophyletic, which highlights substantial incongruence between current taxonomy and evolutionary history inferred from molecular data. Within *Bathynellidae*, the subfamily Bathynellinae forms a well-supported clade C7 of Laurasian genera (*Morimotobathynella*, *Altainella*, *Antrobathynella*, *Pacificabathynella* and European *Bathynella* spp.), mirroring the similar pattern seen in the *Parabathynellidae* family (*Iberobathynella*-group). The well-supported clade 8 includes the subfamily Gallobathynellinae that are distributed in both North America (*Hobbsinella*) and Eurasia (*Gallobathynella, Paradoxicamousella* and *Vejdovskybathynella*). The Indian genus *Indobathynella* that was recovered as sister to Gallobathynellinae supports its closer affinity. This study supports its placement in Gallobathynellinae instead of Bathynellinae (see §5). Although Bathynellinae and Gallobathynellinae are each recovered as geographically restricted clades, their deep divergence and the placement of additional lineages outside these groups suggest that the family *Bathynellidae*, as it is currently defined, does not represent a single natural evolutionary lineage. The sister-group relationship between *Indobathynella* and Gallobathynellinae further highlights the complex evolutionary history of bathynellids and suggests an ancient biogeographic connection between Indian and Laurasian lineages.

One of the key biogeographic insights from this study is the recovery of clades C5 and C6, which are distributed across Australasia, India and Africa, suggesting their Gondwanan origin. However, the long-term isolation of the Indian subcontinent and Australia during their northward drift after breaking away from Gondwana may have created ideal conditions for species to evolve independent evolutionary trajectories in subterranean freshwater lineages. For example, all three known Indian genera (*Indobathynella*, *Serbanibathynella* and *Camachobathynella* (not included in this study)) and Australian genera (*Anguillanella*, *Muccanella*, *Pilbaranella* and *Fortescuenella*) of the family *Bathynellidae* are endemic to their respective countries. This scenario is particularly evident in the divergence-time estimates for *Serbanibathynella*, which falls in the early Cretaceous period, when India was fully separated from the Antarctica–Australia landmass. The strict endemism of Indian bathynellid genera further reinforces the biogeographic significance of the Indian plate, which is recognized as ‘India vera’, representing the largest and oldest region of differentiation for India's original floras and faunas [15]. Together, these patterns suggest that the present-day distributions of Gondwanan bathynellid lineages are best explained by a combination of ancient vicariant events and subsequent long-term isolation, with limited opportunity for post-separation dispersal.

The recovery of clade C5, represented here by the genus *Parvulobathynella*, is consistent with a classic Gondwanan distribution pattern previously documented for this lineage [52,53]. Close relatives of *Parvulobathynella* are known from Africa and South America, although they have not yet been documented from Australia. In our phylogeny, clade C5a corresponds to *Leptobathynellidae* and comprises exclusively Indian taxa (*Parvulobathynella* spp.), recovered as sister to a group of African genera (C5b) currently assigned to *Parabathynellidae*. The deeper divergence among *Leptobathynellidae* (C5a), African genera (C5b) and the Australian Queenslandbathynellinae (C5c), dated to the Early Permian (approx. 263 mya), further indicates that these lineages originated well before the fragmentation of Pangaea and probably experienced prolonged evolutionary isolation within an intact supercontinent.

Previous studies have recovered Queenslandbathynellinae as sister to Austrobathynellinae [37], a relationship that might have been expected here as well. However, the inclusion of Indian taxa in the present global phylogeny resulted in Austrobathynellinae forming a sister relationship with the Indian genus *Serbanibathynella*. Although this grouping receives strong Bayesian support (*PP* = 1.0), support from ML-based analyses is weak (*UFBoot* = 63; *SH-aLRT* = 85), indicating substantial uncertainty. Low bootstrap values generally reflect limited or conflicting phylogenetic signal, suggesting that this sister relationship may represent an artefact of data structure, model assumptions or sparse sampling rather than a robust evolutionary affinity. Under this interpretation, *Serbanibathynella* may not be closely related to Austrobathynellinae, and the previously inferred sister relationship between Austrobathynellinae and Queenslandbathynellinae [37] may still be supported once additional data are incorporated.

In the current study, the results suggest that the present distribution of bathynellacean lineages is probably explained by plate-tectonic-mediated vicariance; however, this scenario should be considered tentative and a working hypothesis. There are a few gaps in the work that need to be bridged to further fortify this hypothesis. The current phylogenetic framework is limited by the absence of molecular data from many key regions, particularly South America and many parts of Africa. This incomplete taxonomic and geographic coverage may not only lead to biased estimates of divergence times, but also limit the feasibility of conducting comprehensive global biogeographic reconstructions. Therefore, this study did not implement formal biogeographic model testing (e.g. DEC, DEC + J). Second, the molecular data used here are very limited; ideally, a phylogenomic approach needs to be implemented to derive a robust and well-supported phylogeny and time tree for this ancient group.

In the present study, divergence-time estimates for the Bathynellacean group are subject to several inherent limitations. As demonstrated by Brown and Smith [54], marginal prior distributions in Bayesian divergence dating arise from interactions among fossil calibrations, user-specified priors, tree priors and clock models, rather than reflecting individual prior specifications in isolation. In addition, divergence-time estimates can be further influenced by taxon sampling, gene region sampling, the number of molecular markers and the inclusion of deep or shallow-level fossil calibrations [55]. Consequently, different analytical configurations can yield substantially different temporal estimates, even with the same molecular data. Although such considerations are central to robust molecular dating, exploring the effects of alternative parameterizations on divergence-time estimates for bathynellaceans lies beyond the scope of the present study. Future studies would therefore benefit from explicitly assessing the sensitivity of Bathynellacean divergence-time estimates to alternative parameter configurations, in order further to evaluate the robustness of the inferred temporal patterns.

## 5. Taxonomic implications

Camacho *et al.* [32,56,57] highlighted the need to revise the generic status of *Hexaiberobathynella* and *Paraiberobathynella* in relation to the broader *Iberobathynella* group (see clade 1, figure 4), despite observable morphological differences between the genera. This taxonomic decision is primarily informed by recent molecular phylogenetic analyses [32,56,57], which reveal that these genera do not form distinct evolutionary lineages. However, this taxonomic reassignment is based on a limited number of genetic markers, and therefore, until comprehensive multilocus datasets are available, caution should be exercised in permanently revising taxonomic classifications. In clade 2, although Schminke [39] proposed a close relationship between *Atopobathynella* and *Chilibathynella*, our molecular analyses do not support this hypothesis; instead, *Chilibathynella* shows closer affinities with the cosmopolitan genus *Hexabathynella*. Likewise, a potential relationship between *Atopobathynella* and *Kimberleybathynella*, suggested by Cho *et al.* [58], Bandari *et al.* [59] and Perina *et al.* [38], remains unresolved even after the inclusion of two Indian *Atopobathynella* species. In our analyses, *Kimberleybathynella* is also recovered as nested within the *Atopobathynella* clade, rendering *Atopobathynella* paraphyletic with respect to *Kimberleybathynella*, albeit with weak to moderate support (*UFBoo*t = 33; *SH-aLRT* = 77), consistent with previous morphological [59] and molecular [38] studies. Given the limited taxon sampling and molecular marker coverage, we refrain from proposing formal taxonomic changes at this stage. Resolving these relationships will require expanded sampling of genera, additional loci, coalescent-based species-tree analyses, topology testing and a comprehensive reassessment of morphological characters. Until such data are available, the phylogenetic placement and evolutionary relationships of these genera should be regarded as provisional and will be addressed in a subsequent, focused taxonomic revision.

Of the five South Korean genera analysed in this study, *Eobathynella* and *Hangangbathynella* show a close evolutionary relationship with Southeast Asian taxa *Paraeobathynella* and *Eobathynella* (clade C3), which points to an origin linked to eastern or southeastern Asian crustal blocks. By contrast, the remaining South Korean genera (*Allobathynella*, *Arisubathynella* and *Nipponbathynella*) are more closely related to European and North American taxa (clade C1), which implies a distinct Laurasian ancestry. This dual pattern indicates the presence of two distinct geological lineages within the South Korean terrain, corroborated by geological evidence indicating that the Korean Peninsula is composed of multiple terranes. Such a scenario suggests that subterranean freshwater lineages in Korea did not arise from a single colonization event, but instead reflect the amalgamation of faunas associated with different continental fragments or palaeogeographic domains. Given the extremely limited dispersal capacity of bathynellaceans, long-distance dispersal is unlikely to account for these patterns, favouring instead a vicariant explanation linked to tectonic assembly and terrane accretion. Together, these results highlight the Korean Peninsula as a biogeographic transition zone that has acted as a repository for multiple ancient lineages, underscoring the importance of integrating phylogenetic, biogeographic and geological data to understand the complex evolutionary history of East Asian subterranean biodiversity.

The clade C5a, corresponding to *Leptobathynellidae* and represented exclusively by Indian taxa (*Parvulobathynella* spp.), was recovered as sister to a group of African genera (*Cteniobathynella*, *Racovitzaibathynella* and *Haplophallonella*) that are currently assigned to *Parabathynellidae*. This relationship, which is strongly supported across phylogenetic inference methods, indicates that current family-level classifications do not fully reflect evolutionary history and suggests that *Parabathynellidae*, as presently defined, may be polyphyletic. Although the present aim of the study is not to propose any formal taxonomic revisions, these results imply that the diagnostic characters traditionally used to distinguish *Leptobathynellidae* and *Parabathynellidae* may be homoplastic or insufficient to capture deep evolutionary divergences. Based on the molecular evidence presented here, we tentatively consider the African genera *Cteniobathynella*, *Racovitzaibathynella* and *Haplophallonella* to be more closely allied with *Leptobathynellida*e than with *Parabathynellidae*, pending further evaluation. Moreover, the placement of this Indian–African assemblage as sister to Queenslandbathynellinae, albeit with low support across analytical frameworks, suggests that several lineages currently assigned to different families share a common ancestry that predates the fragmentation of Gondwana. Collectively, these findings underscore the need for a reassessment of family boundaries within *Bathynellacea*, integrating molecular phylogenetic evidence with expanded taxon sampling and comparative morphological data in a total-evidence framework to achieve a stable and evolutionarily informed classification.

The genus *Serbanibathynella* was established by Ranga Reddy and Schminke (2005) and subsequently assigned to the *Gallobathynella* group by Coineau and Camacho [29]. Later, Camacho [47] treated this genus as *incertae sedis*, and Perina *et al.* [38] suggested that it may warrant recognition as a separate subfamily. In the present study, we re-evaluated the detailed examination of all available morphological characters of two *Serbanibathynella* species (supplementary material, table S6), which revealed that they do not conform to the updated diagnostic keys for the four currently recognized subfamilies proposed by Perina *et al.* [38]. Moreover, repeated field efforts failed to recollect the type species, *Serbanibathynella primaindica* Ranga Reddy and Schminke, 2005 [60], which precludes the formal erection of a new subfamily (Serbanibathynellinae), despite substantial genetic divergence at the *18S* rRNA locus supporting its evolutionary distinctiveness. Phylogenetically, the unexpected recovery of *Serbanibathynella* as sister to Austrobathynellinae (clade C6), albeit with weak support under ML-based analyses, further indicates that the genus cannot be confidently accommodated in any of the four currently recognized Bathynellacean subfamilies. This result underscores either the need to erect a new subfamily or to undertake a thorough revision of subfamily boundaries within *Bathynellidae*. Resolving these classification and relationship issues will require expanded sampling of taxa from India, Africa, Australia and Asia, along with the rediscovery of missing type material, and the explicit integration of molecular and morphological evidence within a total-evidence framework. Until such data are available, taxonomic changes involving *Serbanibathynella* and allied lineages should be regarded as tentative, and existing classifications should be retained as a working hypothesis rather than a definitive evolutionary framework.

Although *Bathynellidae*, as currently defined, is paraphyletic, the subfamilies Bathynellinae (C7) and Gallobathynellinae (C8) are consistently recovered as reciprocally monophyletic. The Indian genus *Indobathynella* is recovered as sister to Gallobathynellinae (*PP* = 0.9; *UFBoot* = 98; *SH-aLRT* = 97), challenging its previous taxonomic placements. Ranga Reddy and Totakura [41] provisionally assigned *Indobathynella* to *Bathynellidae* based on plesiomorphic characters, while explicitly noting that it might represent an as-yet undescribed family. Subsequently, Coineau and Camacho [29] associated the genus with the *Gallobathynella* group, although it was later treated as *incertae sedis* [47]. Our molecular phylogenetic analyses, along with the pronounced genetic divergence observed at the *18S* rRNA locus (data not given), underscore the evolutionary distinctiveness of *Indobathynella* and suggest that it may warrant recognition of a separate subfamily to accommodate its morphological variability (supplementary material, table S6). Nevertheless, given the current limitations in molecular marker coverage, we refrain from proposing formal taxonomic changes at this stage. A comprehensive reassessment of the taxonomic status of *Indobathynella*, integrating expanded molecular datasets with detailed morphological analyses, is currently underway. Until such analyses are completed, the placement of *Indobathynella* within Gallobathynellinae should be regarded as the most parsimonious working hypothesis rather than a definitive taxonomic resolution.

## 6. Conclusion

The deep phylogenetic divergences recovered in this study suggest that the major families of *Bathynellacea* have an ancient origin, probably extending back to the Late Palaeozoic era (Late Carboniferous to Early Permian), confirming previous estimates. This study is consistent with previous hypotheses that the global distribution and limited dispersal capacity of *Bathynellacea* [61] place their ancestral lineage no later than the Upper Palaeozoic [31,62–64], with a distribution correlated to plate tectonic movements and geographic/geological events that occurred on different temporal scales [65,66]. Analysis of the time-calibrated tree reveals that some of the earliest diverging lineages near the root are distributed across continents formerly part of Gondwana and Laurasia. This pattern supports a dual origin scenario, with distinct Gondwanan and Laurasian lineages emerging independently, unlike in previous hypotheses [27,30,31], attributed to a Laurentian origin. As far as its biogeography concerns, this study supports that Bathynellacean diversification was closely tied to major tectonic events, beginning with the assembly of Pangaea in the Permian and followed by vicariant divergence during the break-up of the supercontinent. The strict subterranean lifestyle of bathynellaceans, characterized by ecological specialization and low dispersal capacity, further supports vicariance as the most parsimonious explanation for their current distribution patterns, rather than recent or long-distance dispersal. However, it must be noted that marine regressions/transgressions, as well as recent vicariant events, such as captures of river heads, faults and alpine orogeny, might have also shaped the current distribution of species. Overall, this study reinforces the value of *Bathynellacea* as a model for understanding deep-time biogeography and the consequences of plate-tectonic-mediated vicariance in their current distribution.

## Supplementary Material



## Data Availability

As per the journal's guidelines, we, the authors, have deposited all relevant data generated in the present study in the Zenodo repository, where it is publicly available under the assigned DOI [67]. We further confirm that all data generated for this study are original, primary data produced by the authors and were not obtained from secondary sources. Supplementary material is available online [68].
