# Supplementary material for: Deep history of an ancient subterranean crustacean: origins of the Bathynellacea (Syncarida)
Source: Proc Biol Sci. 2026 Oct 28;293(2081):20260943. doi: 10.1098/rspb.2026.0943 (PMC13714382; doi:10.1098/rspb.2026.0943)
Supplement: Supplementary file 1 [file rspb.2026.0943.zip › rspb.2026.0943/rspb20260943_si_001.rtf]

SUPPLEMENTARY MATERIAL FROM
Deep History of an Ancient Subterranean crustacean: Origins of the Bathynellacea (Syncarida)
Shabuddin Shaik^1^, K Praveen Karanth
Centre for Ecological Sciences, Indian Institute of Science, Bangalore 560012, India.
^1^ Corresponding Author: shabu.biologist@gmail.com
Published in:  Proceedings of the Royal Society B: Biological Sciences
DOI: https://doi.org/10.1098/rspb.

Materials and Methods 
Taxon sampling and study areas: Approximately 300 samples were collected during 2022–2024 from porous and alluvial aquifers associated with various rivers, streams, domestic wells, and farm bores, with a focus on type localities. Samplings were collected using a plankton net made of bolting silk (mesh size: 70 ìm) cloth. The principal study area includes specific regions within the deltaic zone of the Krishna-Godavari Basin in southeastern peninsular India (Fig. 2).
A total of 117 specimens were studied, representing 11 previously described species from four genera, along with four newly identified habrobathynellid species. All collected samples were immediately preserved in 90–100% ethanol and stored at -20°C for further analysis. In this study, DNA was extracted from the abdomen of the individual specimens, and the rest of the appendages (cephalic, thoracic, and pleotelson) were dissected in glycerol under a binocular stereozoom microscope at ×90 magnification and prepared on permanent slides. Anatomical examinations were performed using an oil immersion lens (×100) with a Zeiss interference microscope equipped with a UCA condenser, IC objective prism, and ×1–2magnification changer. Material is deposited in the Centre for Ecological Science (CES) at the Indian Institute of Science. Additional permanent slide preparations and alcohol-preserved whole specimens are in the first author's personal collections for further study. Species-level identification of all individuals from the four genera was carried out following the taxonomic descriptions provided by Ranga Reddy & Totakura (2012a), Bandari et al. (2017), Shaik & Ranga Reddy (2018), and Shaik (2019). 
Molecular Study: Genomic DNA was extracted from whole specimens using the DNeasy Blood & Tissue Kit (Qiagen GmbH, Hilden, Germany), following the manufacturer's protocol. Partial sequences of two gene markers — one nuclear (18S, ~1800 bp) and one mitochondrial (COI, ~700 bp) were amplified via Polymerase Chain Reaction (PCR) and subsequently sequenced. The primers used are listed in Table 7. PCR reactions were performed in a 25 ìL volume containing 12.5 ìL AmpliWin 2X PCR Master Mix, 1 ìL of each primer (0.2 ìM), 8.5 ìL ultrapure water, and 2 ìL of DNA template. Amplification conditions are provided in the Table 3. PCR reactions were carried out using a Mastercycler Gradient (Eppendorf, Hamburg, Germany). PCR products were visualized by gel electrophoresis on a 1.5% agarose gel stained with Diamond Nucleic Acid Dye (Promega, USA). Finally, purification of PCR products was performed using Exo SAP-IT (USB, Amersham, UK) with incubation at 37°C for 45 minutes, followed by enzyme inactivation at 80°C for 15 minutes. The purified PCR product was then sequenced in both directions using a Big Dye Terminator Kit (Applied Biosystems Inc., Foster City, USA). The obtained raw chromatograms were manually verified and edited using Geneious version 10.0.9 (Kearse et al., 2012). Complementary strands were also sequenced and checked against each other for all the samples to ensure accuracy. The consensus sequences were then generated and compared against GenBank database entries to confirm that the obtained sequences were of bathynellacean origin and free from contamination. PCR sequencing was outsourced to Barcode Biosciences (Bangalore, India).
A total 106 published sequences of the 18S and 94 COI gene sequences were obtained from GenBank, representing species from all biogeographic realms except the Neotropical realm (Fig. 3) and encompassing three recognized families (Camacho et al., 2002, 2018a, 2018b, 2021b; Guzik et al., 2008; Ji et al., 2021; Ji & Min, 2022, 2023; Perina et al., 2024). Additional sequences from members of Anaspidacea (Hopel et al., 2023; Lerner & Lucas, 2023) and Stomatopoda (Ahyong, 2005) were included as out-group taxa. These sequences were combined with data generated in this study and the final alignment was subjected to downstream analyses. Furthermore, four fossil calibrations were incorporated into the divergence time analysis. Accession numbers for all published sequences are provided in Table 4 and concatenated data matrix is included as part of the supplementary information.

Phylogenetic analyses: Sequence alignments were performed using MUSCLE (Edgar, 2004) with default parameters, as implemented in MEGA 11 (Tamura et al., 2021). The resulting alignments were manually inspected, and for the COI gene, translated amino acid sequences were examined to verify the correct reading frame and to ensure the absence of stop codons. Maximum likelihood (ML) and Bayesian inference (BI) methods were employed to reconstruct the phylogeny for two genes separately, and no evidence of mito-nuclear discordance was detected, therefore, the gene datasets were concatenated and analyzed together. Twelve species from Anaspidacea and three species from Stomatopoda were included as outgroup taxa for both ML and BI analyses (Table 3). IQ-TREE phylogenomic software online (Nguyen et al., 2015) was used to build an ML tree partitioned by gene, choosing the automatic substitution model option via ModelFinder (Kalyaanamoorthy et al., 2017) (Table. 2). Node support was assessed using ultrafast bootstrapping (UFBoot) (Hoang et al., 2018) and the recommended SH-aLRT test (Shimodaira–Hasegawa approximate likelihood ratio test) (Guindon et al., 2010). The Bayesian analysis was performed in MrBayes 3.2.2 (Ronquist et al., 2012) with the appropriate best-fit model using the Markov chain Monte Carlo approach. All parameters were unlinked, and four chains were run simultaneously for 300 million generations with two independent runs, sampling every 1000^th^ generation. Convergence was assessed using the program Tracer v.1.7.2 (Rambaut et al., 2018) to determine whether estimated sample size (ESS) values were >200. A burn-in of 25% was chosen, and a maximum clade credibility tree was constructed from the remaining post burn-in trees.
Divergence Time Estimation: Bathynellaceans are widely distributed across the globe, however, because of their small size, fragile bodies, and concealed habitats, they do not have a fossil record. Additionally, their anatomy is relatively simple, lacking prominent, sclerotized structures, making it unlikely for taxonomically diagnostic features to be preserved through the normal fossilization process. In the absence of appropriate fossils within the ingroup, we calibrated our phylogenetic tree using four fossil representatives within Eumalacostraca. Ursquilla yehoachi (F1) is placed between Squillidae and Parasquillidae (Stomatopoda), and was used as a distant outgroup to root the phylogenetic tree. The Triassic fossil Anaspidites antiques (F2) was used to calibrate the Anaspidesidae clade, which is considered the immediate sister lineage to the ingroup Bathynellacea. This placement is supported by morphological evidence for syncarid monophyly (Richter & Scholtz, 2001), although molecular investigations (Camacho et al., 2002) did not support a sister connection between Anaspidacea and Bathynellacea, but rather with Euphausiacea (Hopel et al., 2023; Bernot et al., 2023). The oldest known syncarid Palaecaridacea fossil, Palaeocaris retractata (F3) is used to calibrate the syncarida clade. Finally, the oldest crown Eumalacostracan fossil, Palaeopalaemon newberryi (F4) was used to constrain the root age of the tree. For all four calibrations, the minimum fossil ages were applied as hard offsets to ensure clade ages did not fall below the minimum fossil age, and lognormal priors were assigned to all four fossil calibrations. We acknowledge that outgroup calibrations introduce uncertainty and can affect the precision of divergence time estimates, they remain the most reliable option for dating ancient lineages lacking ingroup fossil records (Powell et al., 2020).
Divergence time estimation was performed using BEAST v2.7.7 (Drummond & Rambaut, 2007), executed via in XSEDE in CIPRES Science Gateway online server (Miller et al., 2010). The XML input file was generated using BEAUti v2.7.7 (Drummond et al., 2012). The best-fit partitioning scheme and substitution models, as determined by PartitionFinder (Lanfear et al., 2012), were applied to the analysis. An uncorrelated lognormal relaxed clock model and a Yule speciation prior were used. The Markov chain Monte Carlo (MCMC) analysis was run for 150 million generations, sampling every 1,000 steps. Convergence and stationarity were assessed using Tracer v1.7.2 (Rambaut et al., 2018), ensuring all effective sample size (ESS) values exceeded 200. The first 25% of sampled trees were discarded as burn-in, and the remaining trees were summarized using TreeAnnotator v2.7.7 (Rambaut & Drummond, 2024). The resulting maximum clade credibility tree was visualized using FigTree v1.2.2 (Drummond et al., 2012). Following the approaches of Brown and Smith (2018) and Wolfe et al. (2024), we evaluated the relative influence of marginal priors based fossil versus molecular data on the posterior dates. This was accomplished by comparing marginal prior and posterior distributions of calibrated node ages from the resulting .trees files using the getMRCA function in the R package ape. Prior and posterior age distributions were subsequently compared using kernel density estimates in the R software environment to quantify the extent to which molecular data updated the fossil-based marginal priors. In addition, to quantify the degree to which posterior distributions depart from their corresponding marginal priors, we calculated the Kullback–Leibler (KL) divergence.
The taxonomic debate: The systematic position of Bathynellacea within Malacostraca, particularly within Syncarida, has long been one of the most debated issues in crustacean phylogeny. Central to this debate is whether bathynellaceans represent true members of Syncarida or constitute a separate evolutionary lineage, Podophallocarida, as proposed by Serban (1972) and later reaffirmed by Tabacaru & Danielopol (2011).
Early morphological studies (Calman, 1899, 1917; Brooks, 1962), interpreted Bathynellacea as primitive malacostracans, sharing ancestral characters with Anaspidacea, which led to the view that both groups together form Syncarida as a basal eumalacostracan assemblage and possible ancestors to higher Malacostraca. In contrast, Serban (1972) emphasised the pronounced morphological distinctiveness of Bathynellaceans, particularly in male reproductive structures (podophallus), reduced gill structures, and thoracopodal morphology and argued that these differences justified their segregation as Podophallocarida within the infraclass Eonomostraca. This interpretation was subsequently revisited and supported by Tabacaru & Danielopol (2011), who highlighted that key reproductive characters separating Bathynellacea from Anaspidacea weaken the hypothesis of close syncarid affinity.
The first molecular assessment of Bathynellacea, using 16S rDNA (Camacho et al., 2002), provided limited phylogenetic resolution but already raised questions about their stable placement within Syncarida. Later, Jenner et al. (2009), using a four-gene dataset (18S rRNA, 28S rRNA, 16S rRNA, and COI), recovered unstable and conflicting topologies, with Bathynellaceans, alternately grouping with Amphipoda or forming a basal eumalacostracan lineage distinct from Anaspidacea. Recently, Bernot et al. (2023) conducted a large phylogenomic study using total RNA data from 149 transcriptomes and 16 genomes and found that Syncarida is not monophyletic but paraphyletic. Significantly, neither molecular nor combined molecular–morphological analyses recovered Syncarida as a monophyletic group, leading these authors to conclude that the available data were insufficient to resolve eumalacostracan relationships with confidence.
Overall, both morphological and molecular evidence increasingly suggest that Syncarida does not represent a monophyletic group, broadly supporting Serban's (1972) proposal of Podophallocarida for Bathynellacea. However, this classification was not accepted by Schminke (1981), who proposed the Zoea theory, which provides a robust framework for understanding its relationship to other crustacean groups within the superorder Syncarida and interpreted bathynellaceans as highly simplified or secondarily reduced syncarids rather than an independent lineage. Given the persistent lack of phylogenetic resolution and the continued use of Syncarida in comparative studies, bathynellaceans were universally considered, besides being degenerate anaspidaceans or simplified syncarids (Schminke, 1981; Schram, 1986). We hereby provisionally follow Schminke's (1981) interpretation, while recognising that the systematic position of Bathynellacea within Syncarida remains unresolved and requires phylogenomic reassessment.
Calibration strategy: When estimating divergence times in phylogenies, researchers often turn to secondary calibrations (ages derived from previous molecular dating studies) when fossil evidence for the ingroup is unavailable. However, secondary calibrations are prone to systematic error, as they already carry some uncertainty and potential biases of the original analyses. Conversely, out group fossil calibrations, although phylogenetically more distant, are genuine temporal anchors and often yield more reliable results than secondary calibrations (Powell et al., 2020). Therefore, immediate outgroup fossil calibrations perform better than secondary calibrations. For this reason, secondary calibrations should be regarded only as a last resort. In the present study, we relied on immediate outgroup fossils, given the absence of bathynellacean body fossils. To minimize potential biases, we applied several safeguards: (1) We employed multiple independent fossil calibrations rather than a single constraint to improve both accuracy and precision; (2) We used soft bounds with appropriate probability distributions instead of hard constraints; (3) We selected fossils based on robust phylogenetic frameworks (Bernot et al., 2023 & therein; Hegna et al., 2020 ); and best practices (Parham et al., 2012; Wolfe et al., 2016 ); (4) we applied relaxed clock models to accommodate rate variation among lineages.

Table 1. Fossil calibrations used in this study along with their geological age estimates and justification for placement in the phylogenetic analysis
S. No.	Fossil taxon	Higher classification	Node	Min-max age (mya)	
1	Ursquilla yehoachi	Hoplocarida: Squilloidea	Stomatopoda	72-313	
2	Anaspidites antiques	Eumalacostraca: Anaspidesidae	Anaspidesidae	240-521	
3	Palaeocaris retractata 	Eumalacostraca: Palaeocarididae	Syncarida	322-521	
4	Palaeopalaemon newberryi	Eumalacostraca: Palaeopalaemonidae	Root node	358-521	

Fossil 1

Ursquilla yehoachi Remy and Avnimelech, 1955

Phylogenetic justification: The uropod morphology of U. yehoachi confirms its placement within the crown group Verunipeltata, a clade which includes Bathysquilloidea, Erythrosquilloidea, Eurysquilloidea, Gonodactyloidea, Lysiosquilloidea, Squilloidea, and Parasquilloidea. The fossil U. yehoachi exhibits key diagnostic features shared with Squillidae (Squilloidea), such as the presence of prelateral lobes, submedian teeth with fixed apices, and a subquadrate telson (Haug et al., 2013). As the oldest known squilloid fossil, U. yehoachi provides a critical calibration point for estimating divergence times within Squillidae and Parasquillidae (Stomatopoda). In this study, we employed three living members of Squillidae and Parasquillidae to root the phylogenetic tree as a distant outgroup.

Age Justifications: Member of the Mishash Formation in Israel (Hof, 1998). Stratigraphically, these chert deposits fall within the Hoplitoplacenticeras marroti ammonite biozone (Reiss et al., 1986). H. marroti is known to co-occur with smooth-form Baculites sp., which constrains the age to the uppermost part of the early Campanian stage (Lehmann and Murphy, 2001). Based on the spline-fit of interbedded bentonite layers, the base of the Tethyan Baculites sp. has been dated to approximately 79.64 million years ago, with a duration of 0.63 million years (Ogg et al., 2012). Accordingly, the minimum age of U. yehoachi is estimated at 79.01 million years (Wolfe et. al., 2016), and soft maximum constraint is taken based the recent studies of Haug et. al (2013) and Van Der Wal et. al (2017).
Fossil 2 
Anaspidites antiques Chilton, 1929
Phylogenetic justification: The diagnostic mouthparts of the Anaspides antiquus fossil are not preserved, making its precise familial placement within the order Anaspidacea uncertain. As a result, this fossil has not been incorporated into any molecular phylogenetic studies to date. However, we have used A. antiquus as a calibration point for Anaspidesidae members, following the interpretation of Schram (1984). In his study, Schram strongly supported the close resemblance of A. antiquus to extant members of Anaspidines based on key morphological features, including the fusion of the first thoracic segment with the head, the presence of uniramous pleopods, and the absence of an antennal scale.
Age justification: The fossil A. antiquus was discovered in the Hawkesbury Sandstone in New South Wales, Australia (Schram, 1984). Geologically, the Hawkesbury Sandstone sits between two other rock layers—the Narrabeen Group below it and the Wianamatta Group above it (Herbert, 1997). Based on sequence stratigraphy, the Hawkesbury Sandstone belongs to a layer called "Sequence F," which is marked by the presence of Aratrisporites parvispinosus spores (Helby, 1973; Herbert, 1997). These spores indicate age during the Anisian stage of the Middle Triassic period (Herbert, 1997). The upper boundary of the Anisian is dated to around 241.5 million years ago (±1 million years) (Ogg, 2012), meaning A. antiquus must be at least 240.5 million years old (Wolfe et al., 2016).
Fossil 3 
Palaeocaris retractata Calman, 1932
Phylogenetic justification: The order Palaeocaridacea represents the only extinct group within Syncarida, retaining several plesiomorphic features, although it lacks a caudal furca (a derived character). Bathynellaceans share morphological similarities with Palaeocaridacea but are considered even more primitive (Noodt, 1965).To properly constrain the syncarida clade in our phylogenetic analysis, we incorporated the oldest known syncarid fossil, Palaeocaris retractata, from the Manning Canyon Formation in the USA. This taxon serves as a key reference for assessing early syncarid evolution.
Age Justifications: The fossil P. retractata was recovered from the upper section of the Manning Canyon Formation, which has been dated as the earliest Bashkirian at its top (Ahern and Fielding, 2021). Stratigraphically, this formation dated the Morrowan age (Early Pennsylvanian) of marine deposits as determined by conodont (Webster et al., 1984). The associated flora from the Manning Canyon Shale has been assigned to the lowermost Namurian B (Nelson and Tidwell, 1987). Given the direct stratigraphic association of P. retractata with this floral assemblage, the fossil is most likely of Early Pennsylvanian (Morrowan) age (Schultze, 1990). This temporal framework constrains the fossil P. retractata cannot being younger than approximately 322 million years (Lerner and Lucas, 2023).
Fossil 4 

Palaeopalaemon newberryi whitfield, 1880

Phylogenetic justification: The Eumalacostraca clade encompasses Stomatopoda, Peracarida, Syncarida, and Eucarida. Its monophyly is well supported by combined morphological and molecular phylogenetic analyses (Wills et al., 2009; Lee et al., 2013; Oakley et al., 2013). Thylacocephalans represent an enigmatic group of arthropod fossils, with a stratigraphic range extending from the Silurian to the Cretaceous (Haug et al., 2014; Schram, 2014). The Jurassic thylacocephalan Dollocaris ingens Van Straelen, 1923, was found to be a stem eumalacostracan by phylogenetic analysis (Legg et al., 2013). However, their malacostracan affinities have been questioned by Haug et al. (2014). Given this taxonomic uncertainty, we have elected to use the oldest unequivocal eumalacostracan fossil, Palaeopalaemon newberryi, to constrain the root age of the sl. eumalacostracan clade in our analyses.
Age Justifications: The fossil Palaeopalaemon newberryi based on specimens discovered from Chagrin Shale Member of the Ohio Shale in northeast Ohio, and the Burlington Shale in Iowa, USA (Feldmann et al., 1978; Schram et al., 1978). The Chagrin Shale is assigned to the late Famennian stage of Late Devonian Period. This dating is based on the presence of a specific type of alga known as Foerstia (Murphy, 1973), which occurs at depths of 40–70 meters below the surface in Ohio outcrops (Feldmann et al., 1978). The upper boundary of the Famennian stage is placed at 358.9 ± 0.4 million years ago, providing a conservative minimum age estimate of 358.5 for this fossil (Wolfe et al., 2016).

Table 2. Partitioning scheme and associated molecular markers used in this study, including substitution models selected for each partition
Gene Marker	Gene partition	IQ Tree	BEAST	
18 S rRNA	1-2712	TN+F+I+R6	TN+F+I+G4	
COI (codon position 1)	2713-3417	GTR+F+I+G4	GTR+F+I+G4	
COI (codon position 2)	2714-3417	GTR+F+I+R3	GTR+F+I+G4	
COI (codon Position 3)	2715-3417	GTR+F+R4	GTR+F+G4

	
Table 3. Thermocycler profile and PCR conditions employed for gene amplification in this study.
Gene Marker	Amplification Programme	Reference	
18 S rRNA	Enzyme activation at 95°C for 5 min, followed by 40 cycles of denaturation at 95°C for 30 s, annealing at 49°C for 30 s, and extension at 72°C for 45 seconds. The final extension step was carried out at 72°C for 10 min.	Perina et al., 2023
	
COI	Enzyme activation at 94°C for 4 min, followed by 35 cycles of denaturation at 94°C for 1 min, annealing at 45°C for 1 min, and extension at 72°C for 1 min. The final extension step was carried out at 72°C for 10 min.	Thakur et al., 2020	


Table 4. List of material used in this phylogenetic study with collection locality details, museum voucher numbers, and genbank accession numbers for each genetic marker. Abbreviations: NA (Not Available); *: Incertae sedis
S. NO.	Operational Taxonomic Unit	Location 	Lat/long/elev 
(m, asl)	**Voucher number
COI/18S	GB-AN
COX 1	GB-AN
18	
1. 	Anaspides clarkei (Outgroup)	Exit Cave, Base camp, Tasmania	43.45708000 S 
146.86774000 E
NA	Ana49/Ana378	KX923373	OQ820212	
2. 	Anaspides driesseni (Outgroup)	Levert Rivulet, Tasmania	42.94592500 S
147.23030000 E
NA	Ana502 /Ana502	OP684743	OQ820218	
3. 	Anaspides eberhardi (Outgroup)	Khazad Dum, Tasmania	42.70999900 S
146.55999800 E
NA	Ana402	NC_084311	OQ820213	
4. 	Anaspides jarmani (Outgroup)
	Hartz Mt. NP, Ladies Tarn, Tasmania	43.23638889 S
146.77072222 E
 NA	Ana25/Ana246	KX923349	OQ820209	
5. 	Anaspides richardsoni (Outgroup)	Mt. Field, Robert Tarn, Tasmania	42.67752778 S
146.57094444 E
NA	Ana6/Ana21	KX923327	OQ820208	
6. 	Anaspides spinulae (Outgroup)	Lake St. Clair, Tasmania	42.11000000 S
146.19777778 E
NA	Ana97/Ana488	KX923389	OQ820217	
7. 	Anaspides swaini (Outgroup)
	Mueller Road, Weld River creeks, Tasmania	42.81047222 S
146.43316667 E
NA	Ana38/Ana590	KX923362	OQ820224
	
8. 	Anaspides tasmaniae (Outgroup)
	Mt. Wellington, Chalet, Tasmania	42.89036111 S 147.23608333 E
NA	Ana1/Ana543	KX923322	OQ820221
	
9. 	Allanspides helonomus (Outgroup)
	Scotts Peak Road, Lake Pedder area Tasmania	42.97100000 S
146.35752778 E
NA	Ana21/Ana127	KX923345	OQ820203
	
10.	Allanspides hickmani (Outgroup)	McPartland Pass, Lake Pedder area, Tasmania	42.85733333 S
146.20308333 E
NA	Ana12/Ana141	KX923336	OQ820206
	
11. 	Faughnia profunda (Outgroup)	Western Indian Ocean	NA	NA	MH168240	MN306282	
12. 	Paranaspides lacustris (Outgroup)	Great Lake, Tods Corner, off western shore, Tasmania	41.95833333 S
146.78338889 E
NA	Ana120/Ana120	MF593632	OQ820202	
13. 	Paranaspides williamsi (Outgroup)	Arthurs Lake, near Pumping station, Tasmania	41.98791667 S
146.86127778 E
NA	Ana134/Ana553	MF593634	OQ820222	
14. 	Squilla empusa (Outgroup)	Atlantic Ocean, North Carolina, USA	NA	NA	MH087673	L81946	
15. 	Squilla rugosa (Outgroup)	Florida, USA	NA	NA	HM138810	HM138898	
BATHYNELLIDAE Grobben, 1905	
16. 	Altainella calcarata 	Aktru River, Altai Mountain, Russia	50.06944 N
87.75028 E
2408	MNCN:ADN 54879	MN258523	MN262081	
17.	Altainella mongoliensis
	Khentii, Mongolia	48.290972 N
110.210286 E
 NA	NNIBRIV118588	PQ032499	PQ037634
	
18. 	Anguillanella callawaensis	CA0006, De Grey River, Western
Australia	-20.64445 N
120.30627 E
NA	WAMC 57258	MF074337	MF042209	
19. 	Antrobathynella stammeri	Ogof Cave, United Kingdon	51.831159 N
-3.686986 E	
NA	MNCN:ADN:54711	MF114307	MF094714	
20	Bathynella ruffoi 	Rienza River, San Sigismondo, Italy	46.81054 N	
11.80044 E	
770	MNCN:ADN:54711	MF443329	MF436214	
21. 	Bathynella cf. ruffoi	Dobbiaco Lake, Italy	46.70682 N
12.21926 E
1260	MNCN:ADN:54719	MF443327	MF436212	
22.	Bathynella cf. ruffoi	Aurino River, Gais, Italy	46.84611 N
11.95019 E
850	MNCN:ADN:54727	MF443328	MF436213	
23.	Bathynella (Lombardobathynella) sp.	Mina Rila, Bulgaria	NA	MNCN AND: 54775	 NA	MN262079	
24.	Bathynella sp. 1	Trubarjeva Jama, Slovenia	46.36142 N	
13.77056 E	
1854	MNCN:ADN:54718	MF114308	MF094715	
25.	Bathynella sp. 2	Kamniška Jama, Slovenia	46.32747 N	
14.59031 E	
592	MNCN:ADN:54668	MF114309	MF094716	
26.	Bathynellidae sp.D3.A
	Dawson Catchment, Queensland, Australia	NA	NA	KX022542
	KX022515
	
27.	Bathynellidae sp.D3.B	Dawson Catchment, Queensland, Australia	-24.43022 N
 150.5390 E
NA	NA	KX022544	KX022517	
28.	Bathynellidae sp.D14.A		Dawson Catchment, Queensland, Australia	NA	NA	  KX022538
	KX022511
	
29.	Bathynellidae sp.D14.B	Dawson Catchment, Queensland, Australia	-24.59395 N
149.9299 E
NA	NA	KX022541	KX022514	
30.	Fortescuenella serenitatis	SV0523, Central Hamersley, Western	-22.24878 S
117.6192 E
NA	WAMC57402	MK135011	MK134941	
31.	Gallobathynella boui 	La Devèze Cave, Courniou, France	43.47455 N	
2.71417 E	
365	MNCN:ADN:54600	KP974146	KP999757	
32.	Gallobathynella tarissei	Limousis Cave, Limousis, France	43.34261 N	
2.41346 E	
440	MNCN:ADN:54592	NA	KP999752	
33.	Hobbsinella edwardensis	Edwards Aquifer (Hays County), Texas, USA	29.88958 N	
-97.93645 E	
177	MNCN:AND: 54637	NA	KP999685	
34.	Hobbsinella gunnisonensis	Lottis creek, Colorado, USA	 38.77145 N
-106.62195 E 
2797 	MNCN:AND: 54886	MN258519	MN262077	
35.	Hobbsinella gunnisonensis	Spring creek, Colorado, USA	38.75418 N
-106.76984 E
2622 	MNCN:AND: 54883 	MN258520	MN262078	
*36.	Indobathynella prehensilis	Bore sample, Gayamvaripalem village, Andhra Pradesh, India	16.117770108 N
80.564083486 E
7	CES:SS:00039	NA	PX832275	
*37.	Indobathynella prehensilis	Bore sample, Narikellapalle village, Andhra Pradesh, India	14.276141427 N
80.081196594 E 
5	CES:SS:00041	PX831718	PX832276	
*38.	Indobathynella socrates	Bore sample, Dantelaboru village, Telangana, India	17.59111055 N
80.836956511 E
55	CES:SS:00018	PX831719		PX832277	
39.	Morimotobathynella koreana	Hongcheon-gun, South Korea	37.68772778 N
127.68348056 E NA	NNIBRIV136387	PQ790059
	PQ789943
	
40.	Morimotobathynella sp.	Pocheon, South Korea.	NA	SJ594-1/SJ593-5	PQ790062	PQ789945	
41. 	Muccanella cundalinensis	CU0285, Pilbara, Western Australia	-20.5471 N	
120.1669 E
NA	WAMC57368 	MN136084	MN149112	
42. 	Pacificabathynella yupik	Kwethluk River, Alaska, USA	60.34520 N
161.089146 W 1268	MNCN:ADN 29963	KP974126	NA	
43.	Paradoxiclamousella fideli 	CO220, Carnero Spring Cave 
Cave, Cantabria, Spain	43.28680 N	
-4.47784 E
156 	MNCN:ADN:29735	JX121252	KC469523	
44.	Paradoxiclamousella fideli	CO.069, Pozo Agua Cave, Asturias, Spain	43.2838 N
-4.52318 E
374	MNCN:ADN:29746	JX121253	KC469524	
45. 	Paradoxiclamousella sp 1	CO.209, Treslajorá Cave, Cantabria, Spain	43.26725 N	
-4.59085 E	
1132	MNCN:DNA:29594	JX121249	JX121235	
46. 	Pilbaranella ethelensis	OB23REG1, Pilbara, Western Australia	-23.39367 N	
119.8213? E
NA	WAMC57460	MF074366	MF042210	
47. 	Queenslandnella spinosa 	Bore EEUN2, East End Queensland, Australia	23.88484167 S
150.97208056 E
NA	WAMC 57521
	  PQ420852
	PQ419283
	
48. 	Serbanibathynella secunda	Bore sample, Modukuru village, Andhra Pradesh, India	16.13937418 N
80.57822991 E
9	CES:SS:00009	PX831727	NA	
49. 	Serbanibathynella secunda	Bore sample, Ravulapalem village, Andhra Pradesh, India	16.776605861 N
81.850481638 E
4	CES:SS:00020	NA	PX832284	
50. 	Serbanibathynella secunda	Bore sample, Kunchanapalli village, Andhra Pradesh, India	16.482217980 N
80.617568638 E
49	CES:SS:00035	NA	PX832285	
51. 	Serbanibathynella sp_Telangana	Bore sample, Dantelaboru village, Telangana, India	17.59111055 N
80.836956511 E
55	CES:SS:00017	PX831728	PX832286	
52. 	Vejdovskybathynella edelweiss 	Ojo Guareña Cave (OG01), Burgos, Spain	43.03188 N	
-3.65821 E	
724	MNCN:DNA:29440	HQ596571	KC469513	
53. 	Vejdovskybathynella sp1	Río Chico Cave, Cantabria, Spain	43.19088 N	
-3.58071 E	
573	MNCN:ADN:29728	JX121244	KC469522	
54. 	Vejdovskybathynella sp2
	Redonda Cave, Burgos, Spain	43.03236 N	
-3.62914 E
668	MNCN:ADN:29524 MNCN:ADN:29920 	KP974141	KP999683	
55. 	Vejdovskybathynella vasconica	Goikoetxe Cave, Vizcaya, Spain	43.36026 N
-2.70195 E	
149	MNCN:ADN:29646
	KC469535	KC469521	
PARABATHYNELLIDAE Noodt, 1965	
56. 	Allobathynella cheongdoensis	Dongchang stream, Cheongdo-Gun, South Korea	35.67444444 N
128.86752778 E
NA	NIBRIV0000904532	OR147939	OR149860	
57. 	Allobathynella hongcheonensis	Hongcheon-gun, South Korea 	37.69930556 N
127.66955556 E	NIBRIV0000900580	OP214603
	OP214786	
58. 	Arisubathynella cheongmiensis	Kyeonggi-Do, Yeoju-Gun, Jumdong-Myon, Dori, South Korea	37.23183333 N
127.71780556 E
NA	NNIBRIV103065	OQ689195	OQ689805	
59. 	Arisubathynella pocheonensis	Imjingang & Hangang rivers, South Korea	38.01285556 N
127.24651389 E
NA	NNIBRIV103063	OQ689200	OQ689810	
60. 	Arkaroolabathynella remkoi	Grindell's Hut, Australia	-30.47716 N
139.21348 E
NA	KMA-2012	NA	JQ446056	
61. 	Arkaroolabathynella robusta	Yaltipena Spring, South Australia, Australia	-31.21939 N	
138.7430 E	
NA	NA	JQ898526	JQ898528
	
62. 	Atopobathynella abelloana	Brockman, Pilbara West Australia, Australia	22.57491661 S
117.18995739 E
 NA	BES10765c_BMR02601	OR252691 	PV463798	
63. 	Atopobathynella beasleyensis 	Brockman, Pilbara, West Australia, Australia 	22.71643683 S
117.32626500 E
NA	BES17988_BMR04606 	OR252772	PV463804	
64. 	Atopobathynella glenayleensis	Carnegie Downs, West Australia, Australia	-25.6685 N	
122.3686 E
NA	NA	NA	JQ446052	
65. 	Atopobathynella hinzeae	Depot Spring, West Australia, Australia	-27.93010 N	
120.0584 E
NA	NA	NA	JQ446051	
66. 	Atopobathynella inopinata	Bore sample, Kunchanapalli village, Andhra Pradesh, India	16.479344130 N
80.624902711 E
20	CES:SS:00045	PX831691	PX832242	
67. 	Atopobathynella jessicae 	Miralga Creek, Pilbara West Australia, Austalia	21.11048175 S
119.19448839 E
NA	WAMC78881_BES8283 	PV432466 	PV463819	
68. 	Atopobathynella lythei	Gudai Darri, Pilbara, West Australia	22.56847083 S
119.10918300 E
NA	BMR03366	PP582328	PP580435	
69. 	Atopobathynella operculata	R. Krishna, Challagariga village, Andhra Pradesh, India	16.796504083 N
80.127908700 E
25	CES:SS:00029	PX831692	PX832243	
70. 	Atopobathynella pagetae	Gudai Darri, Pilbara, West Australia, Australia	22.58467500 S
119.18176083 E
NA	BMR03373	PP582325	PP580432	
71. 	Atopobathynella pervulgata 	Rhodes Ridge, Pilbara West Australia, Australia 	23.11978200 S
119.07862283 E
NA	BES10623_BMR03982 	PV432434 	PV463802	
72. 	Atopobathynella rudini 	Brockman, Pilbara West Australia, Australia	22.57479900 S
117.18615681 E
NA	BES7410_BMR00527 	PV432427 	PV463792	
73. 	Atopobathynella runhami 	 Miralga Creek West Australia, Australia	21.12305850 S
119.20001819 E
NA	WAMC78878_BES8282 	PV432464 	PV463818	
74. 	Atopobathynella shawensis 	 Miralga Creek, Pilbara West Australia, Australia	20.69542928 S
119.33067361 E
NA	WAMC78888_BES8290 	PV432472 	PV463822	
75. 	Atopobathynella yarriensis	Yarrie Ridge, Pilbara Region, West Australia	20.60326111 S
120.33985000 E
NA	WAMC76655	OR129981	OR120345	
76.	Billibathynella sp. 1	Austin Downs, West Australia, Australia	 25.874 S 117.4524 E
NA	BES14245	EU350247	JQ446060	
77.	Billibathynella sp. 2A	Moorarie, West Australia, Australia	-27.41337 N	
117.7112 E
NA	BES14775	JN817401	JQ446059	
78.	Brevisomabathynella cooperi 	Jundee, West Australia, Australia	-26.2827 N	
120.6757 E
NA	BES14301B	EU350254	JQ446065	
79.	Brevisomabathynella uramurdahensis 	Bubble Well, West Australia, Australia	-26.56073 N
120.0408 E
NA	BES11147	JN817407	JQ446073	
80.	Chilibathynella sp.1	Peel River, Australia	-31.08361 N
 150.91167 E
NA	KMA-2019	JN817388	MK554634	
81.	Cteniobathynella boutini	Modern well, Moné, Djougou, Donga, Benin West Africa	9.72573 N
1.84716 E
387	MNCN/ADN 54871	NA	PP621896	
82.	Eobathynella sp.2	CC02_Song Tuy Loa, Vietnam	NA	MNCN ADN 54875	NA	MT822872	
83.	Eobathynella gwangjuensis	Gwangju-si , South Korea	35.12198889 N
126.78368889 E
NA	NNIBRIV90877	ON060623	ON062060	
84.	Eobathynella cf. yeojuensis	Womju-si , South Korea	NA	NNIBRIV90880	ON060624	ON062056	
85.	Habrobathynella ajraoi	Bore sample, Thagarapuvalasa town, Andhra Pradesh, India	17.929348 N
83.4205645 E
6	CES:SS:00047	PX831693	PX832244	
86.	Habrobathynella ernstmayr	R. Kinnerasani, Burgampadu village, Telangana, India	17.63365495 N
80.867976431 E
45	CES:SS:00013	PX831694		PX832245	
87.	Habrobathynella parakrishna	Bore sample, Ravulapalem village, Andhra Pradesh, India	16.776605861 N
81.850481638 E
4	CES:SS:00021	PX831695	PX832247	
88.	Habrobathynella parakrishna	Bore sample, Atreyapuram village, Andhra Pradesh, India	16.837520111 N
81.790914738 E
18	CES:SS:00022	PX831696		PX832248	
89.	Habrobathynella cf.parakrishna	Bore sample, Modukuru village, Andhra Pradesh, India	16.13937418 N
80.57822991 E
9	CES:SS:00010	PX831711	PX832264	
90.	Habrobathynella pseudoindica	R. Krishna, Chamarthi village, Andhra Pradesh, India	16.6570012 N
80.133985 E
25	CES:SS:00026	PX831697	PX832249	
91.	Habrobathynella cf. pseudoindica	Bore sample, Dantelaboru village, Telangana, India	17.59111055 N
80.836956511 E
55	CES:SS:00016	PX831712	PX832265	
92.	Habrobathynella cf. pseudoindica	 R. Krishna, Amaravathi village, Andhra Pradesh, India	16.582623611 N
80.361745277 E
19	CES:SS:00024	PX831713	PX832266	
93.	Habrobathynella schminkei	R. Penna, Nellore town, Andhra Pradesh, India	14.4783951167 N
80.005346825 E
14	CES:SS:00042	PX831698	PX832250	
94.	Habrobathynella schminkei	R. Penna, Nellore town, Andhra Pradesh, India	14.471684480 N
79.991456336 E
8	CES:SS:00043	NA	PX832251	
95.	Habrobathynella schminkei	R. Penna, Nellore town, Andhra Pradesh, India	14.471684480 N
79.991456336 E
8	CES:SS:00044	PX831699	PX832252	
96.	Habrobathynella vaitarani complex	R. Krishna, Ravirala village, Andhra Pradesh, India	16.827834 N
80.114764 E
25	CES:SS:00001	PX831700	PX832253	
97.	Habrobathynella vaitarani complex	R. Krishna, Madipadu village, Andhra Pradesh, India	16.813274 N
80.071942 E
25	CES:SS:00003	PX831702	PX832255	
98.	Habrobathynella vaitarani complex	R. Krishna, Madipadu village, Andhra Pradesh, India	16.813274 N
80.071942 E
25	CES:SS:00004	PX831706	PX832259	
99.	Habrobathynella vaitarani complex	R. Krishna, Ramanapeta village, Andhra Pradesh, India	16.742679 N
80.143858 E
25	CES:SS:00005	PX831707	PX832260	
100.	Habrobathynella vaitarani complex	R. Krishna, Ramanapeta village, Andhra Pradesh, India	16.742679 N
80.143858 E
25	CES:SS:00007	PX831708		PX832261	
101. 	Habrobathynella vaitarani complex	R. Krishna, Ramanapeta village, Andhra Pradesh, India	16.742679 N
80.143858 E
25	CES:SS:00008	PX831709	PX832262	
102.	Habrobathynella vaitarani complex	R. Krishna, Challagariga village, Andhra Pradesh, India	16.796504083 N
80.127908700 E
25	CES:SS:00028	PX831701	PX832254	
103.	Habrobathynella vaitarani complex	R. Krishna, Ginjupalli village, Andhra Pradesh, India	16.8087807 N
80.120518555 E
26	CES:SS:00030	PX831703	PX832256	
104.	Habrobathynella vaitarani complex	R. Krishna, Ginjupalli village, Andhra Pradesh, India	16.8087807 N
80.120518555 E
26	CES:SS:00031	PX831704	PX832257	
105.	Habrobathynella vaitarani complex	R. Krishna, Ginjupalli village, Andhra Pradesh, India	16.8087807 N
80.120518555 E
26	CES:SS:00033	PX831705	PX832258	
106.	Habrobathynella vidua	R. Krishna, Ramanapeta village, Andhra Pradesh, India	16.742679 N
80.14377 E
25	CES:SS:00006	PX831710	PX832263	
107.	Habrobathynella sp. 1_Andhra Prasesh	R. Krishna, Ravirala village, Andhra Pradesh, India	16.827834 N
80.114764 E
25	CES:SS:00002	NA	PX832271	
108.	Habrobathynella sp. 1_Andhra Pradesh	R. Krishna, Kothapalli village, Andhra Pradesh, India	16.6717051 N
80.136742555 E
25	CES:SS:00027	NA	PX832268	
109.	Habrobathynella sp. 1_Andhra Pradesh	R. Krishna, Ginjupalli village, Andhra Pradesh, India	16.8087807 N
80.120518555 E
26	CES:SS:00032	NA	PX832269	
110.	Habrobathynella sp. 1_Andhra Pradesh	R. Krishna, Ravirala village, Andhra Pradesh, India	16.8223339 N
80.114764 E
25	CES:SS:00034	NA	PX832270	
111.	Habrobathynella sp. 2_ Andhra Pradesh	Bore sample, Gayamvaripalem village, Andhra Pradesh, India	16.117770108 N
80.564083486 E
7	CES:SS:00040	PX831716	PX832273	
112.	Habrobathynella sp_Karnataka	Bore sample Udbur village, Karnataka, India	11.945265163 N
76.238185772 E
729	CES:SS:00037	PX831714	PX832267	
113.	Habrobathynella sp. 1_Telangana	Bore sample, Beeramguda Town, Telangana, India	17.533363059 N
78.303590653 E
NA	CES:SS:00036	PX831715	PX832272	
114.	Habrobathynella sp. 2_ Telangana	R. Kinnerasani, Burgampadu village, Telangana, India	17.63365495 N
80.867976431 E
45	CES:SS:00014	PX831717	PX832274	
115.	Habrobathynella _Subadult _AP	Bore sample, Atreyapuram village, Andhra Pradesh, India	16.837520111 N
81.790914738 E
18	CES:SS:00023	NA	PX832246	
116.	Hangangbathynella mihoensis	Seokhwa-ri, Cheongju-si, Chungcheongbuk-do, South Korea	36.63583333 N
127.35750000 E
NA	NIBRIV0000879486	MW429327	ON062081	
117.	Hangangbathynella taechooni	Kyeonggi-Do, Yeoju-Gun, Juemdong- Myeon Do-Ri, Cheongmi stream, South Korea	37.23183333 N
127.71780556 E
NA	NIBRIV0000904534	OR147941	OR149862	
118.	Haplophallonella irenae	Ouet Douar River, Totous, Chad	19.43944 N
17.52972 E
585	MNCN:ADN:29986	NA	KP999766	
119.	Hexabathynella bisetosa 	Rottnest Island West Australia, Australia	32.01275833 S
115.51306944 E
NA	WAMC59249 	OP758838	OP755270	
120.	Hexabathynella sevillaensis	Santiago Grande Cave, Sevilla, Spain	38.03013 N
-5.90429 E
358	MNCN/ADN 29545	HQ659869	KC469526	
121.	Hexabathynella sp. 1	Port Kenny, South Australia, Australia	-33.1564 N	
134.644 E
NA	KMA-2012	JN817387	JQ446049	
122.	Hexaiberobathynella mateusi	MSS Guadarrama Mountain Range, Madrid, Spain	40.81665 N 
-3.84853 E
 1875	MNCN ADN 54699	NA	MT822873	
123.	Iberobathynella asturiensis	Infierno Cave, Asturias, Spain	43.28068 N
-4.99305 E
950	MNCN/ADN 54896	MZ128377	MT874908	
124.	Iberobathynella cf. asturiensis	Infierno Cave, Asturias, Spain	43.28068 N
-4.99305 E
950	MNCN ADN 54900	OL679951	MT822874	
125. 	Iberobathynella burgalensis	 Ojo Guareña Cave (OG53), Burgos, Spain	43.03188 N
-3.65821 E
NA	MNCN ADN 29520	HQ659859	KC469529	
126.	Iberobathynella cantabriensis	CO.276 Cave, Cantabria, Spain	43.28755 N
-4.50909 E
492	MNCN/ADN 29372	HQ659853	KP999704	
127. 	Iberobathynella cornejoensis	Redonda Cave, Burgos, Spain	43.03236 N	
-3.62913 E	
668	MNCN:ADN:29949	KP974108	KP999701	
128. 	Iberobathynella espaniensis	Infierno Cave, Asturias, Spain	43.28068 N
-4.99305 E
 950	MNCN:ADN 54913/COI	OL679960	MT822875	
129. 	Iberobathynella imuniensis	Torca Morteros Cave, Burgos, Spain	43.14786 N
-3.59539 E	
1285	MNCN:ADN 29166	HQ659850	KC469528	
130. 	Iberobathynella cf. imuniensis (cryptic ?)	Torca Calleja-Cueto, Arredondo, Cantabria, Spain	43.2918 N
-3.62869 E
 649	MNCN ADN 54926	MZ128379	MT822876	
131. 	Iberobathynella magna	Infierno Cave, Asturias, Spain	43.2806 N
-4.99305 E
 950	MNCN/ADN 29370/COI	HQ659858 	MT822877	
132.	Iberobathynella ortizi	Rei Cintolo Cave, Lugo, Spain	43.39364 N	
-7.36786 E
409	MNCN/ADN 54616	MF443322	MF436209	
133.	Iberobathynella parasturiensis	CO.209, Treslajorá Cave, Cantabria, Spain	43.26725 N
-4.59085 E
1132	MNCN:ADN:29566	KC469544	KC469530	
134.	Iberobathynella cf. parasturiensis	Infierno Cave, Asturias, Spain	43.28068 N
 -4.99305 E
 950	MNCN:ADN 54897	OL679948	MT822878	
135.	Iberobathynella sp.	Astui Cave, Vizcaya, Spain	43.21756 N	
-2.18939 E
131	MNCN:ADN:29978	KP974110	KP999710	
136.	Iberobathynella sp. 2	CO.209, Treslajorá Cave, Cantabria, Spain	43.26725 N
-4.59085 E	
1132	MNCN:ADN:29573	KP974102	KP999725	
137.	Iberobathynella sp. 3 	CO.069 Cave, Asturias, Spain	43.28383 N	
-4.52318 E	
374	MNCN:ADN:29705	KP974079	KP999713	
138. 	Kimberleybathynella callani 	 Ord River, Kimberley West Australia, Australia	17.64545483 S
127.92779400 E
NA	BMR08553 	PV432454 	NA	
139.	Kimberleybathynella ordensis	Ord River, Kimberley West Australia, Australia	17.59604236 S
127.92874350 E
NA	BMR08554	PV432455 	PV463813	
140. 	Lockyerenella danschmidti	Queensland, Australia	-27.54144 N	
152.3936 E	
NA	NA	KX022561	KX022524	
141.	Montanabathynella pecosensis	Live Oak, Crockett County, Texas, USA	30.74220 N
-101.6739 E 
655	MNCN ADN 54864	NA	MT822881	
142.	Nicolenella sp. 3	Bore YARC102, Yalleen,
Pilbara, West Australia, Australia	NA	WAMC57110	MK546315 	MK554588	
143.	Nicolenella sp. 4	Bore YA233, Yalleen, ,
Pilbara, West Australia, Australia	NA	WAMC57183	MK546336 	MK554608	
144.	Nipponbathynella yeongchunensis	Danyang-gun, South Korea	37.08347778 N
128.48253056 E
 NA	NIBRIV000090594	OR509937	OR507233	
145.	Nipponbathynella gapcheonensis	Daejeon-si, South Korea	37.08347778 N
128.48253056 E
 NA	NIBRIV000090594	OR509940	OR507235	
146.	Notobathynella sp. 1 	Hunter River, Australia 	-32.0064 S
150.5328 E
NA	KMA-2012 isolate PH3	JN817400	NA	
147.	Octobathynella peelensis	Peel River, New South Wales, Australia	-30.9561 N	
150.8017 E
NA	NA	JN817399	JQ446076	
148.	Parabathynella cf. motasi	Poltarica Spring, Slovenia	45.88681 N	
14.7684	 E
272	MNCN ADN 54669	MF443326	MF436211	
149.	Paraeobathynella ratensis	Rat Cave, Thung Song (Kapang), Thailand	8.04672	 N
99.72847 E	
89	MNCN/ADN 54673	MG271865	MG321597	
150.	Paraeobathynella siamensis	Khao Krot Plu Cave, Khanom (Kuanthong), Thailand	9.23944	 N
99.82000 E	
56	MNCN/ADN 54688	MG271872	MG321604	
151.	Paraeobathynella hanjavanitiana	Khao Plu Cave, Thung Song (Khao Ro), Thailand	9.02292	 N
99.57669 E	
45	MNCN/ADN 54692	MG271873	MG321605	
152.	Paraeobathynella sp.	Thien Duong Cave, Quang Binh, Vietnam	NA	MNCN ADN 54876	NA	MT874909	
153.	Paraiberobathynella cf. fagei 1	Higuera Cave, Murcia, Spain	37.97836 N	
-1.50170 E	
496	MNCN:ADN:29666 	KP974028	KP999736	
154.	Paraiberobathynella cf. fagei 2	Pileta Cave, Málaga, Spain	36.69133 N	
-5.26981 E	
729	MNCN:ADN:54581	KP974043	KP999742	
155.	Paraiberobathynella cf. maghrebensis	Well, Nador, Maghreb, Morocco	34.95238 N	
-2.59795 E	
276	MNCN:ADN:29935	KC469542	KC469532	
156.	Racovitzaibathynella beninensis 
	Modern wells (BEN147), Vanhoui, Benin, West Africa	9.565192 N
1.821983 E
388	MNCN/ADN 54873
 	NA	PP621898
	
157.	Racovitzaibathynella dumonti	Ouet Douar River, Totous, Chad	19.43944 N
17.52972 E
585	MNCN:ADN:29982	NA	KP999762	
158.	Texanobathynella aaronswinki	Sesson Creek, Hays County, Texas, USA	29.88996 N
-97.93596 E
 177	MNCN:ADN 54862	MZ128381	MT822879	
159.	Texanobathynella aaronswinki 	South Llano River, Kimble County, Texas, USA	30.45342 N
-99.80060 E
520	MNCN:ADN 54865	NA	MT822880	
160.	Texanobathynella coloradoensis	Colorado River, Bastron County, Texas, USA	30.11187 N
-97.32775 E
94	MNCN:ADN 54867	MZ128382	MT874910	
LEPTOBATHYNELLIDAE Noodt, 1965	
161.	Parvulobathynella distincta	R. Godavari, Andhra Pradesh, India	25.15656 N
92.20717 E
39	MNCN/ADN 54754	MF443330	MF436215	
162.	Parvulobathynella distincta	R. Godavari, Andhra Pradesh, India	25.15656 N
92.20717 E
39	MNCN/ADN 54755	MF443331	MF436216	
163.	Parvulobathynella distincta	R. Godavari, Andhra Pradesh, India	25.15656 N
92.20717 E
39	MNCN/ADN 54757	MF443332	MF436217	
164.	Parvulobathynella distincta	R. Godavari, Andhra Pradesh, India	25.15656 N
92.20717 E
39	MNCN:ADN:54758	MF443333	MF436218	
165.	Parvulobathynella distincta	R. Kinnerasani, Burgampadu village, Telangana, India	17.63365495 N
80.867976431 E
45	CES:SS:00012	PX831720	PX832278	
166.	Parvulobathynella distincta	R. Kinnerasani, Burgampadu village, Telangana, India	17.633461211 N
80.8715150 E
37	CES:SS:00015	PX831721	PX832279	
167.	Parvulobathynella distincta	R. Godavari, Rajahmundry town, Andhra Pradesh, India	17.004449530 N
81.755177588 E
13	CES:SS:00019	PX831722	PX832280	
168.	Parvulobathynella distincta	R. Krishna, Chamarthi village, Andhra Pradesh, India	16.6570012 N
80.133985 E
25	CES:SS:00025	PX831723	PX832281	
169.	Parvulobathynella macrodentata	Bore sample, Modukuru village, Andhra Pradesh, India	16.138633511 N
80.578202661 E
8	CES:SS:00011	PX831724	PX832282	
170.	Parvulobathynella macrodentata	Bore sample, Gayamvaripalem village, Andhra Pradesh, India	16.124253386 N
80.566402841 E
8	CES:SS:00038	PX831725	PX832283	
171.	Parvulobathynella macrodentata	Bore sample, Kunchanapalli village, Andhra Pradesh, India	16.479344130 N
80.624902711 E
20	CES:SS:00046	PX831726	NA	

**Note: The figure named “7_MLTree_Voucher Number” in supplementary data represents the Maximum Likelihood (ML) tree with voucher numbers corresponding to those shown in Figure 4 of the main manuscript. Clade labels (C1, C2, etc.) have been omitted to avoid redundancy, as the tree topology is identical to the annotated ML tree presented in the main manuscript. Voucher numbers are provided alongside each taxon name to facilitate traceability, enabling other researchers to retrieve the exact sequence data used in this study and thereby replicate, verify, or extend the phylogenetic analyses.
Table 5. Number of genera, species and population studied from different countries in the phylogenetic analysis
Bathynellidae	Species studied	Population studied (Countries covered)	
Altainella	02	01 (Russia, Mongolia)	
Anguillanella	01	01 (Australia)	
Antrobathynella	01	01 (UK)	
Bathynella	04	06 (Italy, Bulgaria, Slovenia)	
Bathynellidae sp. (undetermined genus)	04	04 (Australia)	
Fortescuenella 	01	01 (Australia)	
Gallobathynella 	02	02 (France)	
Hobbsinella 	02	03 (USA)	
Indobathynella
Morimotobathynella	02
02	03 (India)
02 (South Korea)	
Muccanella 	01	01 (Australia)		
Pacificabathynella	01	01 (USA)	
Paradoxiclamousella	02	03 (Spain)	
Pilbaranella 	01	01 (Australia)	
Serbanibathynella
Queenslandnella 	02
01	04 (India)
01 (Australia)	
Vejdovskybathynella 	04	04 (Spain)	
Total: 17 genera studied
	33	39 (12)	
Parabathynellidae 			
Allobathynella 	02	02 (South Korea)	
Arisubathynella 	02	02 (South Korea)	
Arkaroolabathynella 	02	02 (Australia)	
Atopobathynella 	14	14 (Australia, India)	
Billibathynella 	02	02(Australia)	
Brevisomabathynella	02	02 (Australia)	
Chilibathynella
Ctenibathynella	01
01	01 (Australia)
01 (Benin)	
Eobathynella 	03	03 (Vietnam, South Korea)	
Habrobathynella	12	22 ( India)	
Hangangbathynella 	02	02 (South Korea)	
Haplophallonella	01	01 (Chad)	
Hexabathynella 	02	02 (Spain, Australia)	
Hexaiberobathynella	01	01 (Spain)	
Iberobathynella
Kimberleybathynella	13
02	14 (Spain)
02 (Australia)	
Lockyerenella	01	01 (Australia)	
Montanabathynella 
Nicolenella	01
02	01 (USA)
02 (Australia)	
Nipponbathynella
Notobathynella 	02
01	02 (South Korea)
01 (Australia)	
Octobathynella 	01	01 (Australia)	
Parabathynella 	01	01 (Slovenia)	
Paraeobathynella 	04	04 (Thailand, Vietnam)	
Paraiberobathynella	03	03 (Spain, Moracco)	
Racovitzaibathynella 	02	02 (Chad)	
Texanobathynella 	02	03 (USA)	
Total: 27 genera studied
	82	94 (11)	
Leptobathynellidae			
Parvulobathynella
	02	(11) India	
Total: 01 genera studied
	02	 11 (1)	
Grand Total: 45 genera studied	117 species	144 populations (over 17 countries)	
			


Table 6. Principal morphological differences among the four recognized subfamilies of Bathynellidae and two Indian genera of incertae sedis (Serbanibathynella and Indobathynella). Abbreviations: AI, antennule; AII, antenna; Md, mandible; Ths, thoracopods; Seg, segments; Endp, endopod; Exp, exopod.
Characters/Taxa	Serbanibathynella	Indobathynella	Bathynellinae	Gallobathynellinae	Austrobathynellinae	Queenslandbathynellinae	
Body size	Small	Reduced	Small to large	Small to large	Medium	Medium	
AI: seg.	7	7	7	6/7	7	7	
AII directed	Perpendicular to AI	Perpendicular to AI	Anterior	Anterior	Anterior	Anterior	
AII: seg.	7	4	7	5-7	7	8	
AII: medial seta on exp.	Absent	Present/absent	Present/absent	Present/absent	Absent	Absent	
Paragnaths: ornamentation	Setae & tooth	Spinules/denticles	Setae & tooth	Setae 	Setae & tooth/claw	Setae & tooth	
Labrum	Smooth/spinule	Smooth	Smooth	Smooth	Dimorphic	Two medial teeth	
Md palp: seg.	3	3	3	1–3	3	3	
Md:claws	2	2	2	1–2	2	3	
Md palp: sexual dimorphism	Absent	Absent	Present/absent	Present/absent	Absent	Absent	
MxI: lateral setae (ornamentation)	2 (barbed or plumose)	3 (smooth)	3 (plumose)	3 (barbed or plumose)	3 (plumose) 	2 (smooth)	
MxI: claws on endite 1	3/4	1	4	4	4	3	
MxII: seg.	4	3	4	4	4	3	
MxII: prehensility	Absent	Present	Absent	Absent	Absent	Absent	
Ths. I-VII: exp. Seg. (no. of setae)	1 (4)	2 (4)	1 (5)	1 (4-5)	1 (4)	1 (3)	
Th. I: endp. Seg.	3	3	4	3/4	4	4	
Ths. II-VII: endp. Seg.	3	2	4	3/4	4	4	
Male Th. VIII: penial region	Absent	1 lobe	2–3 lobes	1 or 2 lobes	1–4 lobes	4 lobes	
Male Th. VIII: endp. Seg.	Absent	Absent	1	1	1/2	2	
Male Th. VIII: exp. Seg.	1 with distinct basis	Fused with basipod	1 with distinct basis	1 with distinct basis	1 with distinct basis	1 with distinct basis	
Male Th. VIII: endp. /exp.	Absent	Absent	Endp. < exp.	Endp. < exp.	Endp. = exp.	Endp. = exp.	
Female Th. VIII: endp. Seg.	If present 1-segmented	No such differentiated structures	1	1	2?	2	
Female Th. VIII: exp. Seg.	1 as fused basexopod	No such differentiated structures 	1	1	Absent?	1	
Pleopod 1: seg.	2	1	2	2	2	2	
Pleopod 2: seg.	Absent	Absent	Present/absent	Absent	Absent	Absent	
Pleopod: sexual dimorphism	Absent	Present	 Absent	Absent	Absent	Absent	
Uropod: sympod spines	5	3	4-8	4	4	6	
Furcal rami: spines	5 without dorsal spine	4+ dorsal spine	4 + dorsal spine	4 + dorsal spine	4 + dorsal spine	2 + dorsal spine	
Furcal rami: setae	Absent	Present	Present/absent	Absent	Absent	Absent	

Table 7. List of primers used in this study
Marker	primer	Sequence	Reference
	
COI
	C1-J-1718F
C1-J-2329R	5ʹ-GGAGGATTTGGAAATTGATTAGTTCC-3ʹ
5ʹ-ACTGTAAATATATGATGAGCTCA-3
	Simon et al. (1994)	
	LCO1490F
HCO2198R	5ʹ-GGTCAACAAATCATAAAGATATTGG-3ʹ
5ʹ-TAAACTTCAGGGTGACCAAAAAATCA-3ʹ
	Folmer et al. (1994)	
18Si
	1F
5R	5ʹ-TACCTGGTTGATCCTGCCAGTAG-3ʹ
5ʹ-CTTGGCAAATGCTTTCGC-3ʹ
	Giribet et al. (1996)	
18Sii
	3F
18Sbi
	5ʹ-5ʹ-GTTCGATTCCGGAGAGGGA-3ʹ
5ʹ-GAGTCTCGTTCGTTATCGGA-3ʹ
	Giribet et al. (1996)
Whiting et al. (1997)	
18Siii
	18Sa2.0
9R	5ʹ-ATGGTTGCAAAGCTGAAAC-3ʹ
5ʹ-GATCCTTCCGCAGGTTCACCTAC-3ʹ	Whiting et al. (1997)
Giribet et al. (1996)	

References
Ahern JP, Fielding CR. 2021 Carboniferous Manning Canyon Formation, northern Utah, USA: a carbonate-mud-dominated cyclothem motif recording the main onset of the late Paleozoic ice age. Sediment. Geol. 418, 105903. (doi:10.1016/j.sedgeo.2021.105903)
Ahyong ST. 2005 Phylogenetic analysis of the Squilloidea (Crustacea: Stomatopoda). Invertebr. Syst. 19, 189–208. (doi:10.1071/IS04037)
Bandari E, Shaik S, Ranga Reddy Y. 2017 A phylogenetic review of the genus Atopobathynella Schminke, 1973 (Crustacea, Malacostraca, Bathynellacea) with three new species from southeastern India. J. Nat. Hist. 51, 2143–2184. (doi:10.1080/00222933.2017.1360528)
Bernot JP, Owen CL, Wolfe JM, Meland K, Olesen J, Crandall KA. 2023 Major revisions in pancrustacean phylogeny and evidence of sensitivity to taxon sampling. Mol. Biol. Evol. 40, msad175. (doi:10.1093/molbev/msad175)
Brooks HK. 1962 On the fossil Anaspidacea, with a revision of the classification of the Syncarida. Crustaceana 4, 229–242.
Calman WT. 1899 On the characters of the crustacean genus Bathynella Vejdovský. J. Linn. Soc. (Zool.) 27, 338–344.
Calman WT. 1917 Notes on the morphology of Bathynella and some allied Crustacea. J. Cell Sci. 61, 489–514.
Camacho AI, Rey I, Dorda BA, Machordom A, Valdecasas AG. 2002 A note on the systematic position of the Bathynellacea (Crustacea, Malacostraca) using molecular evidence. Contr. Zool. 71, 123–129.
Drummond AJ, Rambaut A. 2007 BEAST: Bayesian evolutionary analysis by sampling trees. BMC Evol. Biol. 7, 214. (doi:10.1186/1471-2148-7-214)
Drummond AJ, Suchard MA, Xie D, Rambaut A. 2012 Bayesian phylogenetics with BEAUti and the BEAST 1.7. Mol. Biol. Evol. 29, 1969–1973. (doi:10.1093/molbev/mss075)
Edgar RC. 2004 MUSCLE: multiple sequence alignment with high accuracy and high throughput. Nucleic Acids Res. 32, 1792–1797. (doi:10.1093/nar/gkh340)
Feldmann RM, Osgood RG Jr, Szmuc EJ, Meinke DW. 1978 Chagrinichnites brooksi, a new trace fossil of arthropod origin. J. Paleontol. 52, 287–294. 
Folmer O, Black M, Hoeh W, Lutz R, Vrijenhoek R. 1994 DNA primers for amplification of mitochondrial cytochrome c oxidase subunit I from diverse metazoan invertebrates. Mol. Mar. Biol. Biotechnol. 3, 294–299.
Giribet G, Carranza S, BagunÞa J, Riutort M, Ribera C. 1996 First molecular evidence for the existence of a Tardigrada + Arthropoda clade. Mol. Biol. Evol. 13, 76–84. (doi:10.1093/oxfordjournals.molbev.a025547)
Guindon S, Dufayard JF, Lefort V, Anisimova M, Hordijk W, Gascuel O. 2010 New algorithms and methods to estimate maximum-likelihood phylogenies: assessing the performance of PhyML 3.0. Syst. Biol. 59, 307–321. (doi:10.1093/sysbio/syq010)
Guzik MT, Abrams KM, Cooper SJB, Humphreys WF, Cho JL, Austin AD. 2008 Phylogeography of the ancient Parabathynellidae (Crustacea: Bathynellacea) from the Yilgarn region of Western Australia. Invertebr. Syst. 22, 205–216. (doi:10.1071/IS07033)
Haug C, Briggs DE, Mikulic DG, Kluessendorf J, Haug JT. 2014 The implications of a Silurian and other thylacocephalan crustaceans for the functional morphology and systematic affinities of the group. BMC Evol. Biol. 14, 159. (doi:10.1186/1471-2148-14-159)
Haug C, Kutschera V, Ahyong ST, Vega FJ, Maas A, Waloszek D, Haug JT. 2013 Re-evaluation of the Mesozoic mantis shrimp Ursquilla yehoachi based on new material and the virtual peel technique. Palaeodiversity 6, 1–15. (doi.org/10.26879/340)
Hegna TA, Luque J, Wolfe JM. 2020 The fossil record of the Pancrustacea. In Evolution and biogeography, vol. 8 (eds M Thiel, G Poore). New York, NY: Oxford Academic. (doi:10.1093/oso/9780190637842.003.0002)
Helby R. 1973 Review of Late Permian and Triassic palynology of New South Wales. Geol. Soc. Aust. Spec. Publ. 4, 141–155.
Herbert G. 1997 Sequence stratigraphic analysis of early and middle Triassic alluvial and estuarine facies in the Sydney Basin, Australia. Aust. J. Earth Sci. 44, 125–143. (doi:10.1080/08120099708728302)
Hoang DT., Chernomor O., von Haeseler A., Minh BQ., & Vinh LS. 2018. UFBoot2: improving the ultrafast bootstrap approximation. Mol. Biol. Evol. 35, 518–522. (doi:10.1093/molbev/msx281)
Hof CHJ. 1998 Late Cretaceous stomatopods (Crustacea, Malacostraca) from Israel and Jordan. Contrib. Zool. 67, 257–266. (doi:10.1163/18759866-06704003)
Höpel CG, Yeo D, Ahyong ST, Meier R, Richter S. 2023 First mitochondrial genomes of Anaspidacea (Malacostraca, Crustacea) and the phylogenetic relationships of mountain shrimps (Anaspides Thomson, 1894) and their relatives within Anaspidesidae. J. Crustac. Biol. 43, ruad028. (doi:10.1093/jcbiol/ruad028)
Jenner RA, Dhubhghaill CN, Ferla MP, Wills MA. 2009 Eumalacostracan phylogeny and total evidence: limitations of the usual aspects. BMC Evol. Biol. 9, 1–20.
Ji SJ, Lee CW, Min GS. 2021 A new species of Hangangbathynella (Crustacea, Bathynellacea, Parabathynellidae) from South Korea. ZooKeys 1046, 143–155. (doi: 10.3897/zookeys.1046.66141)
Ji SJ, Min GS. 2022 DNA barcoding of Allobathynella yecheonensis (Bathynellacea: Parabathynellidae) from interstitial groundwater, South Korea. Anim. Syst. Evol. Divers. 38, 283–286. (doi:10.5635/ASED.2022.38.4.030)
Ji SJ, Min GS. 2023 Two new species of Nipponbathynella Schminke, 1973 from South Korea, with a brief discussion on Korean parabathynellid genera (Crustacea, Bathynellacea). Diversity 15, 1052. (doi:10.3390/d15101052)
Kalyaanamoorthy S, Minh BQ, Wong TK, Von Haeseler A, Jermiin LS. 2017 ModelFinder: fast model selection for accurate phylogenetic estimates. Nat. Methods 14, 587–589. (doi:10.1038/nmeth.4285)
Kearse M, Moir R, Wilson A, Stones-Havas S, Cheung M, Sturrock S, et al. 2012 Geneious Basic: an integrated and extendable desktop software platform for the organization and analysis of sequence data. Bioinformatics 28, 1647–1649. (doi:10.1093/bioinformatics/bts199)
Lanfear R, Calcott B, Ho SY, Guindon S. 2012 PartitionFinder: combined selection of partitioning schemes and substitution models for phylogenetic analyses. Mol. Biol. Evol. 29, 1695–1701. (doi:10.1093/molbev/mss020)
Lee MS, Soubrier J, Edgecombe GD. 2013 Rates of phenotypic and genomic evolution during the Cambrian explosion. Curr. Biol. 23, 1889–1895. (doi:10.1016/j.cub.2013.07.055) 
Legg DA, Sutton MD, Edgecombe GD. 2013 Arthropod fossil data increase congruence of morphological and molecular phylogenies. Nat. Commun. 4, 2485. (doi:10.1038/ncomms3485)
Lehmann J, Murphy MA. 2001 A new placenticeratid ammonite from the Albian (Lower Cretaceous) of California and the inferred history of some placenticeratid and engonoceratid ammonites. N. Jb. Geol. Paläontol. Monatsh. 2001 (6), 343–358. (doi:10.1127/njgpm/2001/2001/343)
Lerner AJ, Lucas SG. 2023 Oldest record of the fossil syncarid (Crustacea) Palaeocaris from the Carboniferous Manning Canyon Formation, Utah, USA. Geobios 77, 37–44. (doi: 10.1016/j.geobios.2023.03.003)
Miller MA, Pfeiffer W, Schwartz T. 2010 Creating the CIPRES Science Gateway for inference of large phylogenetic trees. In 2010 Gateway Computing Environments Workshop (GCE), pp. 1–8. IEEE. (doi:10.1109/GCE.2010.5676129)
 Murphy JL. 1973 Protosalvinia (Foerstia) zone in the Upper Devonian sequence of eastern Ohio, northwestern Pennsylvania, and western New York. Geol. Soc. Am. Bull. 84, 3405–3410.
Nelson CR, Tidwell WD. 1987 Brodioptera stricklandi n. sp. (Megasecoptera: Brodiopteridae), a new fossil insect from the Upper Manning Canyon Shale Formation, Utah (Lowermost Namurian B). Psyche 94, 309–316. (doi:10.1155/1987/52032)
Nguyen LT, Schmidt HA, Von Haeseler A, Minh BQ. 2015 IQ-TREE: a fast and effective stochastic algorithm for estimating maximum-likelihood phylogenies. Mol. Biol. Evol. 32, 268–274. (doi:10.1093/molbev/msu300)
Noodt W. 1965 Natürliches System und Biogeographie der Syncarida (Crustacea, Malacostraca). Gewässer und Abwässer 37(38), 77–186.
Oakley TH, Wolfe JM, Lindgren AR, Zaharoff AK. 2013 Phylotranscriptomics to bring the understudied into the fold: monophyletic Ostracoda, fossil placement, and pancrustacean phylogeny. Mol. Biol. Evol. 30, 215–233. (doi:10.1093/molbev/mss216) 
Ogg JG, Hinnov LA, Huang C. 2012 Cretaceous. In The Geologic Time Scale (eds. F.M. Gradstein, J.G. Ogg, M. Schmitz, G.M. Ogg), pp. 793–853. Elsevier.
Parham JF, Donoghue PCJ, Bell CJ, Calway TD, Head JJ, Holroyd PA, Inoue JG, Irmis RB, Joyce WG, Ksepka DT, et al. 2012 Best practices for justifying fossil calibrations. Syst. Biol. 61, 346–359. (doi:10.1093/sysbio/syr107)
Perina G, Camacho A, Cooper SJB, Floeckner S, Blyth AJ, Saccò M. 2023 An integrated approach to explore the monophyletic status of the cosmopolitan genus Hexabathynella (Crustacea, Bathynellacea, Parabathynellidae): two new species from Rottnest Island (Wadjemup), Western Australia. Syst. Biodivers. 21, Article 2151662. (doi:10.1080/14772000.2022.2151662) 
Perina G, Camacho AI, White NE, Morgan L, Lawrie A, Floeckner S, Guzik MT. 2024 Unveiling hidden Bathynellidae (Crustacea: Bathynellacea) diversity in Australia: an integrated study reveals remarkable diversity and a new subfamily from Queensland. Zool. J. Linn. Soc. 202, zlae151. (doi:10.1093/zoolinnean/zlae151)
Powell CLE, Waskin S, Battistuzzi FU. 2020 Quantifying the error of secondary vs. distant primary calibrations in a simulated environment. Front. Genet. 11, 252. (doi:10.3389/fgene.2020.00252)
Rambaut A, Drummond AJ, Xie D, Baele G, Suchard MA. 2018 Software for systematics and evolution. Syst. Biol. 67, 901–904. (doi:10.1093/sysbio/syy032)
Rambaut A, Drummond AJ. 2024 TreeAnnotator v1.7.0. Available at: http://beast.bio.ed.ac.uk
Ranga Reddy Y, Totakura VR. 2012a A new phreatic species of the genus Parvulobathynella (Malacostraca: Bathynellacea) from southeastern India, along with an updated key to the species. J. Crustac. Biol. 32, 871–882. (doi:10.1163/193724012X637444)
Reiss Z, Almogi‑Labin A, Lewy Z, Moshkovitz S. 1986 Biostratigraphic datums in the Senonian of Israel. Proc. K. Ned. Akad. Wetensch. Ser. B. Palaeontol. Geol. Phys. Chem. Anthropol. 89, 95–104.
Richter S, Scholtz G. 2001 Phylogenetic analysis of the Malacostraca (Crustacea). J. Zool. Syst. Evol. Res. 39, 113–136. (doi:10.1046/j.1439-0469.2001.00185.x)
Ronquist F, Huelsenbeck JP, Teslenko M. 2012 Draft MrBayes version 3.2 manual: tutorials and model summaries. Available at: http://brahms.biology.rochester.edu/software.html (accessed 2025).
Schminke HK. 1981 Adaptation of Bathynellacea (Crustacea: Syncarida) to life in the interstitial ('Zoea theory'). Int. Rev. Ges. Hydrobiol. 66, 575–637.
Schram FR, Feldmann RM, Copeland MJ. 1978 The Late Devonian Palaeopalaemonidae and the earliest decapod crustaceans. J. Paleontol. 52, 1375–1387. See https://www.jstor.org/stable/1303941
Schram FR. 1984 Fossil Syncarida. Trans. San Diego Soc. Nat. Hist. 20(13), 189–246. (doi:10.5962/bhl.part.29006)
Schram FR. 1986 Crustacea. Oxford, UK: Oxford University Press, 606 pp.
Schram FR. 2014 Family level classification within Thylacocephala, with comments on their evolution and possible relationships. Crustaceana 87, 340–363. (doi:10.1163/15685403-00003289)
Schultze HP. 1990 A new acanthodian from the Pennsylvanian of Utah, USA, and the distribution of otoliths in gnathostomes. J. Vertebr. Paleontol. 10, 49–58.
Serban E. 1972 Bathynella (Podophallocarida, Bathynellacea). Trav. Inst. Spéléol. «E. Racovitza» 11, 11–224.
Shaik S, Ranga Reddy Y. 2018 On the genus Habrobathynella Schminke, 1973 (Crustacea, Malacostraca, Bathynellacea), with description of three new species from India. Zootaxa 4492, 1–72. (doi:10.11646/zootaxa.4492.1.1)
Shaik S. 2019 A new Indobathynella species from an Indian cave. The first cavernicolous Bathynellidae (Syncarida: Bathynellacea) from South-Eastern India. Zootaxa 4565, 345–360. (doi:10.11646/zootaxa.4565.3.3)
Simon C, Frati F, Beckenbach A, Crespi B, Liu H, Flook P. 1994 Evolution, weighting and phylogenetic utility of mitochondrial gene sequences and a compilation of conserved polymerase chain reaction primers. Ann. Entomol. Soc. Am. 87, 651–701. (doi.org/10.1093/aesa/87.6.651).
Tabacaru I, Danielopol DL. 2011 Essai d'analyse critique des principales hypothèses concernant la phylogénie des Malacostracés (Crustacea, Malacostraca). Trav. Inst. Spéléol. «E. Racovitza» 50, 87–119.
Tamura K, Stecher G, Kumar S. 2021 MEGA11: Molecular Evolutionary Genetics Analysis version 11. Mol. Biol. Evol. 38, 3022–3027. (doi:10.1093/molbev/msab120)
Thakur SS, Lone AR, Tiwari N, Yadav S. 2020 Exploring new records of Eutyphoeus sp. (Haplotaxida: Octochaetidae) from Garo Hills, Meghalaya, north‑eastern India with use of DNA barcodes. Mitochondrial DNA Part A 31, 265–272. (doi:10.1080/23802359.2020.1820631)
Van Der Wal C, Ahyong ST, Ho SY, Lo N. 2017 The evolutionary history of Stomatopoda (Crustacea: Malacostraca) inferred from molecular data. PeerJ 5, e3844. (doi:10.7717/peerj.3844)
Webster GD, Brenckle P, Gordon Jr M, Lane H, Langenheim Jr R, Sanderson G, Tidwell W. 1984 The Mississippian‑Pennsylvanian boundary in the eastern Great Basin. Neuvième Congrès International de Stratigraphie et de Géologie du Carbonifère, Comptes Rendu, 406–418.
Whiting MF, Carpenter JC, Wheeler QD, Wheeler WC. 1997 The Strepsiptera-problem: phylogeny of the holometabolous insect orders inferred from 18S and 28S ribosomal DNA sequences and morphology. Syst. Biol. 46, 1–68. (doi:10.1093/sysbio/46.1.1)
Wills M, Jenner R, Dhubhghaill C. 2009 Eumalacostracan evolution: conflict between three sources of data. Arthropod Syst. Phylogeny 67, 71–90. (doi:10.3897/asp.67.e31689)
Wolfe JM, Ballou L, Luque J, Watson-Zink VM, Ahyong ST, Barido-Sottani J, et al. 2024 Convergent adaptation of true crabs (Decapoda: Brachyura) to a gradient of terrestrial environments. Syst. Biol. 73, 247–262. (doi.org/10.1093/sysbio/syad066)
Wolfe JM, Daley AC, Legg DA, Edgecombe GD. 2016 Fossil calibrations for the arthropod Tree of Life. Earth‑Sci. Rev. 160, 43–110. (doi:10.1016/j.earscirev.2016.06.008)
